# EGFR-TKIs or EGFR-TKIs combination treatments for untreated advanced EGFR-mutated NSCLC: a network meta-analysis

**DOI:** 10.1186/s12885-024-13168-8

**Published:** 2024-11-12

**Authors:** Ao Liu, Xiaoming Wang, Lian Wang, Han Zhuang, Liubo Xiong, Xiao Gan, Qian Wang, Guanyu Tao

**Affiliations:** Department of Respiratory Medicine, Chengdu BOE Hospital, Chengdu, Sichuan Province 610000 China

**Keywords:** EGFR-TKI, Non-small cell lung cancer, Osimertinib, Lazertinib, Amivantamab

## Abstract

**Background:**

Epidermal growth factor receptor (EGFR) tyrosine kinase inhibitors (TKIs) and EGFR-TKI combination treatments have become the standard first-line treatments for EGFR-mutated non-small cell lung cancer (NSCLC) patients. However, the best option has yet to be determined. This study compares the efficacy and safety of various first-line EGFR-TKI monotherapies and combination treatments for advanced EGFR-mutated NSCLC.

**Methods:**

We searched PubMed, Embase, the Cochrane Central Register of Controlled Clinical Trials databases, and several international conferences to identify randomized controlled trials reporting on first-line EGFR-TKI treatments for patients with advanced EGFR-mutated NSCLC. The study quality was assessed using the revised tool for risk of bias in randomized trials. The efficacy and safety outcomes of the included treatments were compared by network meta-analysis based on a frequentist approach.

**Results:**

We identified 26 trials (8,359 patients) investigating 14 treatment groups, including first, second, and third-generation EGFR-TKIs and their combination treatments. Osimertinib plus chemotherapy and lazertinib plus amivantamab showed the highest efficacy in improving progression-free survival. New third-generation EGFR-TKIs demonstrated comparable efficacy to osimertinib alone but did not surpass it. Subgroup analyses revealed slight variation in treatment efficacy based on mutation types and patient demographics. Combination treatments were associated with a higher incidence of adverse events.

**Conclusion:**

These results reveal that osimertinib plus chemotherapy and lazertinib plus amivantamab are superior first-line options for patients with advanced EGFR-mutated NSCLC. However, these combinations are associated with higher adverse event rates.

**Supplementary Information:**

The online version contains supplementary material available at 10.1186/s12885-024-13168-8.

## Introduction

Lung cancer is the leading cause of cancer-related deaths in men and the second leading cause in women worldwide [1]. In 2022, the estimated number of new lung cancer cases and deaths were 2.5 million and 1.8 million, respectively. Due to the lack of identifiable clinical symptoms in early lung cancer, the majority of patients are diagnosed at an advanced stage [2]. 

Non-small cell lung cancer (NSCLC) comprises over 85% of lung cancer cases [3, 4], with epidermal growth factor receptor (EGFR) mutations occurring in 9–46% of NSCLC cases [5, 6]. For individuals with advanced EGFR-mutated NSCLC, targeted therapy utilizing EGFR tyrosine kinase inhibitors (TKIs) serves as a cornerstone of first-line treatment. These therapies have demonstrated efficacy in enhancing progression-free survival, improving health-related quality of life, and minimizing severe treatment-related side effects compared to traditional chemotherapy [7–15]. However, resistance to TKIs inevitably develops within approximately 8–18 months [16–18]. 

To counter resistance and improve patient survival rates, the oncology research community is actively exploring combinations of EGFR-TKIs with other therapeutic modalities, including chemotherapies, monoclonal antibodies, immunotherapies, and pathway inhibitors, aiming for a synergistic effect in combating NSCLC [19]. Recent evidence has highlighted the superior progression-free survival outcomes of first-line treatments such as osimertinib plus chemotherapy and amivantamab plus lazertinib compared to osimertinib monotherapy [20, 21]. Additionally, the emergence of new third-generation EGFR-TKIs, particularly those developed in Asia, has shown encouraging efficacy for EGFR-mutated NSCLC [22–25]. 

Despite extensive randomized controlled trials and meta-analyses assessing the efficacy and safety of EGFR-TKIs for advanced NSCLC patients with EGFR mutations, the optimal therapeutic strategy remains controversial, particularly in light of specific clinicopathological characteristics of patients and emerging evidence [26–35]. In our study, we conducted a network meta-analysis of randomized controlled trials to evaluate the efficacy and safety of various EGFR-TKI and EGFR-TKI combination therapies for advanced NSCLC with EGFR mutations. We aimed to identify the optimal clinical choice for patients and subgroups based on the primary EGFR mutations (exon 19 deletions and exon 21 L858R mutation) and clinicopathological details.

## Methods

### Protocol and registration

This Network Meta-analysis (NMA) followed the Preferred Reporting Items for Systematic Reviews and Meta-analyses for Network Meta-analysis (PRISMA-NMA) reporting guideline [36]. This protocol has been registered at PROSPERO under registration number CRD42023452689.

### Literature search

We searched PubMed, Embase, and the Cochrane Central Register of Controlled Trials to collect relevant articles with English language restrictions from inception to July 10, 2024, that assessed first-line treatment in patients with advanced EGFR-mutated NSCLC. Additionally, the abstracts of the American Society of Clinical Oncology (ASCO), European Society of Medical Oncology (ESMO), and World Conference on Lung Cancer (WCLC) meetings were also searched to include complete and updated outcomes (2023–2024). The reference lists from all included studies were screened to identify potentially relevant evidence. The detailed search strategy is presented in Supplementary eTable 1.

### Study inclusion and exclusion criteria

We included Phase II/III randomized controlled trials that met the following criteria: (a) Trials that enrolled patients with histologically confirmed advanced (stage III/IV/relapsed) NSCLC with EGFR mutations; (b) Trials that compared one EGFR-TKI versus another or EGFR-TKI combination treatment as first-line treatment; (c) Trials that reported on at least one of the following clinical outcomes: progression-free survival, overall survival, objective response rate, or adverse events (AEs); (d) Published in English. We excluded studies not adhering to the inclusion criteria and other exclusion criteria: (a) Trials reporting results for patients with EGFR-mutated NSCLC from a subgroup analysis; (b) Trials comparing therapies not approved by any food and drug administration; (c) Trials comparing therapy protocols not recommended by guidelines.

### Data extraction

Two authors (LX and LW) independently extracted the following data from each relevant study: study ID, first author, publication year, number of patients, patient characteristics, treatments, and outcomes. To mitigate potential assessment bias by investigators, survival data were preferably extracted by an independent review facility. Treatment-related adverse events were preferred. If not specified as treatment-related, all adverse events were extracted. We consulted ClinicalTrials.gov and other available sources to ensure access to the most recent and complete data. When essential data were unclear or unreported, study authors and pharmaceutical companies were contacted. Any discrepancies in the data extraction process were resolved through consensus discussion when necessary.

Monotherapy with pemetrexed showed inferior efficacy compared to pemetrexed combined with platinum and is not usually recommended as a first-line treatment [37]. Therefore, EGFR-TKI plus pemetrexed was excluded from our analysis [38]. Given the equivalent efficacy among first-generation EGFR-TKIs, we merged gefitinib, erlotinib, and icotinib into a single treatment modality [39, 40]. 

### Quality assessment

Two authors (HZ and XG) independently participated in the quality assessment, and disagreements were resolved by discussion. The quality of the evidence was assessed using the revised tool for risk of bias in randomized trials (RoB 2 tool) [41]. 

### Outcome measure

We synthesized all direct and indirect evidence to compare different treatments regarding efficacy and safety. Hazard ratios (HR) were reported for survival outcomes (progression-free and overall survival). Risk ratios (RR) were reported for binary outcomes (objective response rate and AEs) along with corresponding 95% confidence intervals (CIs). If the 95% CIs of an HR or RR did not include 1, it indicated a significant association. The primary outcome of this study was progression-free survival. Secondary outcomes were overall survival, objective response rate, and AEs.

### Statistical analysis

We conducted a network meta-analysis using the R package ‘netmeta’ (version 1.2-0), adopting a frequentist approach with a random effects consistency model [42, 43]. In our NMA, treatments were ranked based on P-scores. It’s important to note that P-scores rank treatments based on their point estimates and standard errors. Still, they do not directly quantify the certainty of one treatment being superior to another [44]. The reliability and validity of the networks were appraised by addressing inconsistencies and heterogeneity across the comparative studies of various treatments [45]. We employed Cochran’s Q test to evaluate inconsistency within our network model and to identify contributing factors. The net-split function assessed the concordance between direct and indirect evidence [46, 47]. Heterogeneity between studies was quantified using the REML method [48, 49]. A τ^2 value less than 0.1 is usually viewed as very low heterogeneity, from 0.1 to 0.5 as moderate, and over 0.5 as high heterogeneity. Subgroup analyses were conducted, focusing exclusively on data from distinct patient demographics. Small-study effects were examined using funnel plots and statistically assessed with the Begg’s test. A two-sided *P* < 0.05 was considered statistically significant [50]. 

## Results

### Characteristics of the studies

**Fig. 1:**
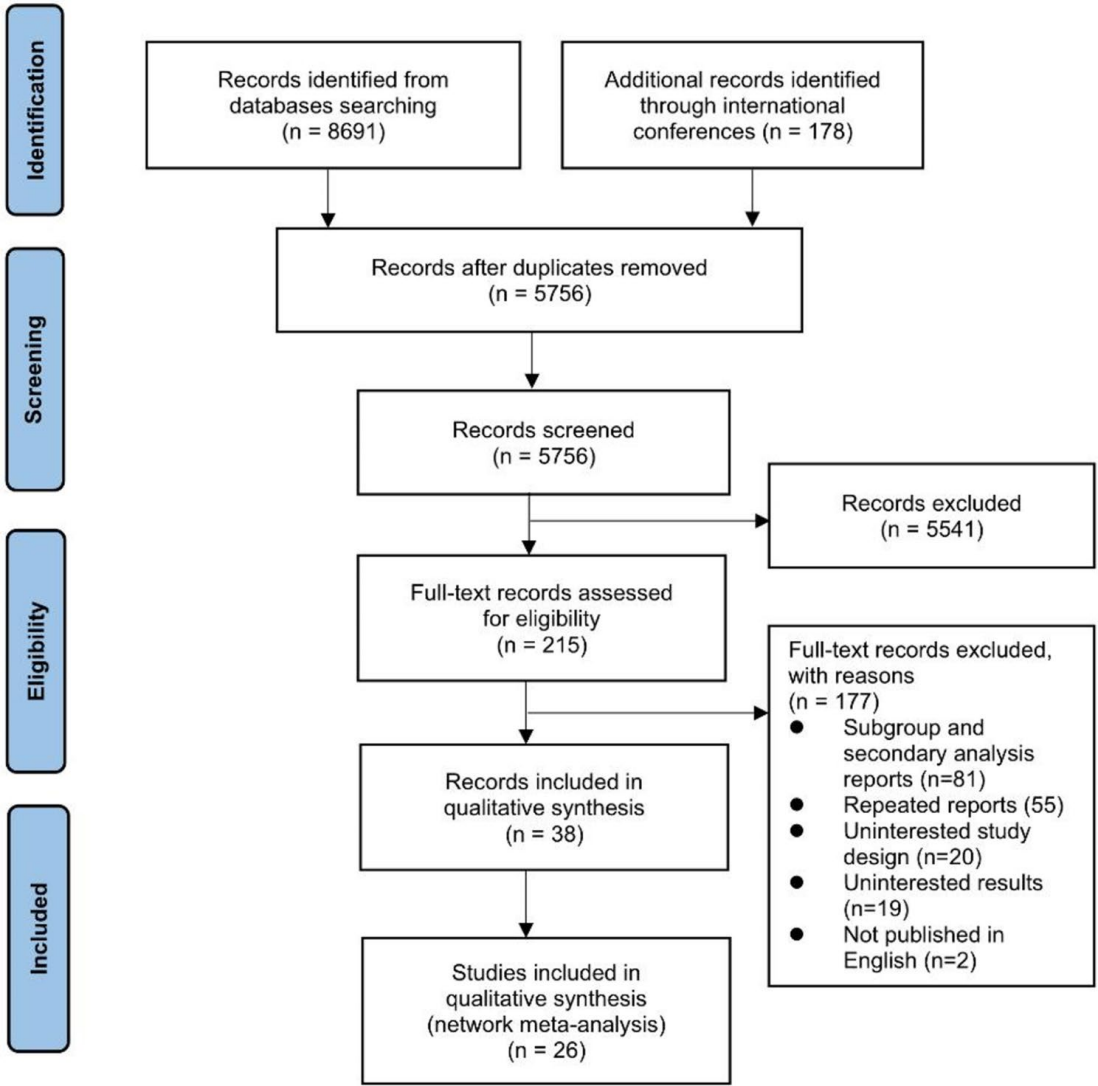
PRISMA flowchart

The study selection process for this network meta-analysis is depicted in Fig. 1. We included a total of 26 trials, encompassing 8,359 patients. The analysis covered 14 groups based on the therapeutic agents and combinations used. These therapeutic agents are first-generation EGFR-TKIs (FGTKI): gefitinib, erlotinib, and icotinib; second-generation EGFR-TKIs: afatinib (Afa) and dacomitinib (Dac); third-generation EGFR-TKIs: osimertinib (Osi), befotertinib (Bef), furmonertinib (Fur), lazertinib (Laz), and aumolertinib (Aum); chemotherapy with pemetrexed plus platinum (PemP); antiangiogenic agents (AntV): apatinib, bevacizumab, and ramucirumab; the anti-EGFR antibody: cetuximab (Cet) and EGFR-MET bispecific antibody: amivantamab (Ami). The foundational characteristics of the included studies are summarized in Table 1. The risk of bias assessment for each study is detailed in eFigure 1.

**Table 1:** The basic characteristics of the included studies

Study year	Phase	Country	Interventions	Sample size	Median age	Sex Female/ Male	EGFR mutation 19DEL	EGFR mutation L858R	Reported outcomes
JO25567 2014	III	Japan	Erlotinib 150 mg QD + bevacizumab 15 mg/kg Q3W	75	67	45/30	40	35	Progression-free survival, overall survival, objective response rate, grade ≥ 3 AEs
			Erlotinib 150 mg QD	77	67	51/26	40	37	
LUX-Lung7 2016	II	International	Afatinib 40 mg QD	160	63	91/69	93	67	Progression-free survival, overall survival, objective response rate, grade ≥ 3 AEs
			Gefitinib 250 mg QD	159	63	106/53	93	65	
ARCHER1050 2017	III	International	Dacomitinib 45 mg QD	227	62	146/81	134	92	Progression-free survival, overall survival, objective response rate, grade ≥ 3 AEs
			Gefitinib 250 mg QD	225	61	125/100	133	92	
FLAURA 2018	II	International	Osimertinib 80 mg QD	279	64	178/101	175	104	Progression-free survival, overall survival, objective response rate, grade ≥ 3 AEs
			Gefitinib 250 mg QD or erlotinib 150 mg QD	277	64	172/105	174	103	
RELAY 2019	III	International	Erlotinib 150 mg QD + ramucirumab 10 mg/kg Q2W	224	65	141/83	123	99	Progression-free survival, overall survival, objective response rate, grade ≥ 3 AEs
			Erlotinib 150 mg QD	225	64	142/83	120	105	
NEJ026 2019	III	Japan	Erlotinib 150 mg QD + bevacizumab 15 mg/kg Q3W	112	67	71/41	56	56	Progression-free survival, overall survival, objective response rate, grade ≥ 3 AEs
			Erlotinib 150 mg QD	112	68	73/39	55	57	
Stinchcombe et al. 2019	II	US	Erlotinib 150 mg QD + bevacizumab 15 mg/kg Q3W	45	63	31/12	NR	NR	Progression-free survival, overall survival, objective response rate
			Erlotinib 150 mg QD	43	63	31/14	NR	NR	
Xu et al. 2019	II	China	Icotinib 125 mg TID	90	59	57/33	51	38	Progression-free survival, overall survival, objective response rate
			Icotinib 125 mg TID + (pemetrexed 500 mg/m2 + carboplatin AUC = 5 Q3W (6 cycles)), then icotinib and pemetrexed maintenance	89	61	66/23	52	37	
SWOGS1403 2020	II	International	Afatinib 40 mg QD + cetuximab 500 mg/m2 Q2W	83	66	59/24	53	30	Progression-free survival, overall survival, objective response rate, grade ≥ 3 AEs
			Afatinib 40 mg QD	85	66	53/32	54	31	
NEJ009 2020	III	Japan	Gefitinib 250 mg QD + (pemetrexed 500 mg/m2 + carboplatin AUC = 5 Q3W (4–6 cycles)), then gefitinib and pemetrexed maintenance	170	65	114/56	93	69	Progression-free survival, overall survival, objective response rate, grade ≥ 3 AEs
			Gefitinib 250 mg QD	172	64	108/64	95	67	
Noronha et al. 2020	II	India	Gefitinib 250 mg QD + (pemetrexed 500 mg/m2 + carboplatin AUC = 5 Q3W) (4 cycles), then gefitinib and pemetrexed maintenance	174	54	86/88	107	60	Progression-free survival, overall survival, objective response rate, grade ≥ 3 AEs
			Gefitinib 250 mg QD	176	56	83/93	109	60	
ACE-Lung 2021	II	France	Afatinib 40 mg QD	59	68	43/16	NR	NR	Progression-free survival, overall survival, objective response rate, grade ≥ 3 AEs
			Afatinib 40 mg QD + cetuximab 500 mg/m2 Q2W	58	63	41/17	NR	NR	
CTONG1706 2021	III	China	Gefitinib 250 mg QD + apatinib 500 mg QD	157	57	91/66	81	74	Progression-free survival, overall survival, objective response rate, grade ≥ 3 AEs
			Gefitinib 250 mg QD	156	60	94/62	83	73	
CTONG1509 2021	III	China	Erlotinib 150 mg QD + bevacizumab 15 mg/kg Q3W	157	57	97/60	82	75	Progression-free survival, overall survival, objective response rate, grade ≥ 3 AEs
			Erlotinib 150 mg QD	154	59	96/58	79	75	
WJOG9717L 2022	II	Japan	Osimertinib 80 mg QD	61	66	32/23	36	25	Progression-free survival, overall survival, objective response rate, grade ≥ 3 AEs
			Osimertinib 80 mg QD + bevacizumab 15 mg/kg Q3W	61	67	37/24	35	26	
AENEAS 2022	III	China	Aumolertinib 110 mg QD	214	59	134/80	140	74	Progression-free survival, overall survival, objective response rate, grade ≥ 3 AEs
			Gefitinib 250 mg QD	215	62	135/80	141	74	
BEVERLY 2022	III	Italy	Erlotinib 150 mg QD + bevacizumab 15 mg/kg Q3W	80	66	52/28	44	34	Progression-free survival, overall survival, objective response rate, grade ≥ 3 AEs
			Erlotinib 151 mg QD	80	68	50/30	44	32	
FURLONG 2022	III	China	Furmonertinib 80 mg QD	178	59	116/62	91	87	Progression-free survival, overall survival, objective response rate, grade ≥ 3 AEs
			Gefitinib 250 mg QD	179	60	111/68	92	87	
LASER301 2023	III	International	Lazertinib 240 mg QD	196	67	132/64	121	75	Progression-free survival, overall survival, objective response rate, grade ≥ 3 AEs
			Gefitinib 250 mg QD	197	64	119/78	122	75	
GAPBRAIN 2023	III	China	Gefitinib 250 mg QD + (pemetrexed 500 mg/m2 + cisplatin 75 mg/m2 or nedaplatin 80 mg/m2 Q4W) (4–6 cycles), then gefitinib and pemetrexed maintenance	80	55	44/36	NR	NR	Progression-free survival, overall survival, objective response rate, grade ≥ 3 AEs
			Gefitinib 250 mg QD	81	56	43/38	NR	NR	
AvaTa 2023	II	Korea	Erlotinib 150 mg QD + bevacizumab 15 mg/kg Q3W	64	50	44/20	37	27	Progression-free survival, overall survival, objective response rate, grade ≥ 3 AEs
			Erlotinib 150 mg QD	63	53	40/23	37	26	
Lu et al. 2023	III	China	Befotertinib 75 to 100 mg QD	182	60	110/72	117	65	Progression-free survival, overall survival, objective response rate, grade ≥ 3 AEs
			Icotinib 125 mg TID	180	58	108/72	117	63	
FLAURA2 2023	III	International	Osimertinib 80 mg QD + (carboplatin AUC = 6 + pemetrexed 500 mg/m2 Q3W) (≤ 6 cycles), then osimertinib and pemetrexed maintenance	279	61	173/108	172	106	Progression-free survival, overall survival, objective response rate, grade ≥ 3 AEs
			Osimertinib 80 mg QD	279	62	170/109	169	109	
OSIRAM-1 2023	II	Japan	Osimertinib 80 mg QD + ramucirumab 10 mg/kg Q2W	57	70	35/24	37	23	Progression-free survival, overall survival, objective response rate
			Osimertinib 80 mg QD	58	67	35/26	36	24	
RAMOSE 2023	II	US	Osimertinib 80 mg QD + ramucirumab 10 mg/kg Q3W	93	nr	66/27	64	29	Progression-free survival, overall survival, objective response rate, grade ≥ 3 AEs
			Osimertinib 80 mg QD	46	nr	33/13	32	14	
MARIPOSA 2024	III	International	Lazertinib 240 mg QD + amivantamab 1050 or 1400 mg QW	429	64	275/154	257	171	Progression-free survival, overall survival, objective response rate, grade ≥ 3 AEs
			Osimertinib 80 mg QD	429	63	251/158	257	172	

AE = adverse event; NR = not reported; AUC = area under the concentration-time curve; QD = once a day; BID = twice a day; TID = three times a day; QW = once every week; Q2W = once every two weeks; Q3W = once every three weeks

### Network geometry and synthesis of results

#### Network meta-analysis in advanced EGFR mutated NSCLC

**Fig. 2:**
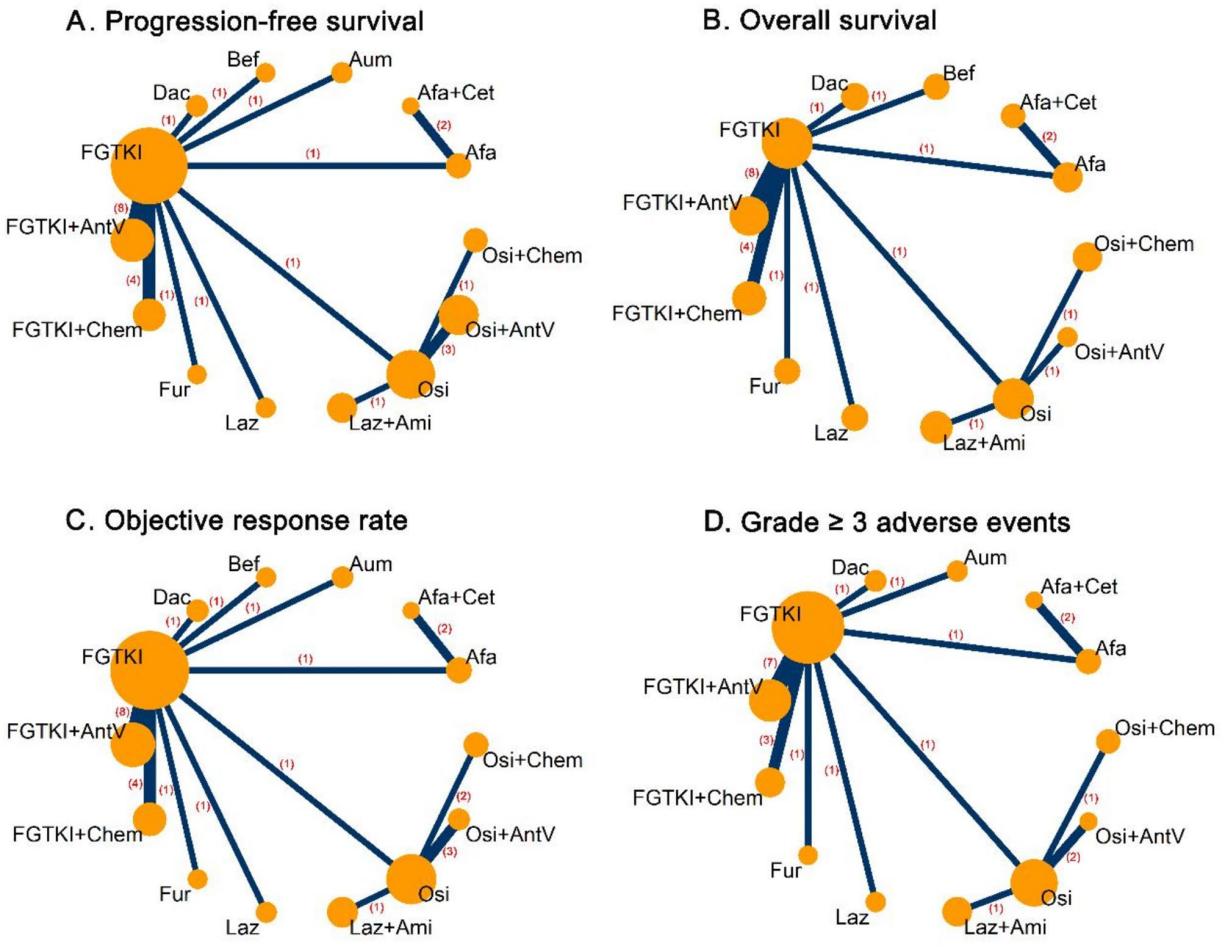
Network diagrams of the network meta-analysis. **A**-**D**: progression-free survival, overall survival, objective response rate and grade ≥ 3 adverse events in patients with advanced EGFR mutated NSCLC. FGTKI = first-generation EGFR-TKIs; Afa = afatinib; Dac = dacomitinib; Osi = osimertinib; Bef = befotertinib; Fur = furmonertinib; Laz = lazertinib; Aum = aumolertinib; Ami = amivantamab; Cet = cetuximab; Chem = chemotherapy; AntV = antiangiogenic agents

The network geometry for each outcome is shown in Fig. 2: progression-free survival and objective response rate analyses included 14 treatment groups from 26 studies with a total of 7801 patients and 7778 patients, respectively; overall survival included 13 treatment groups from 23 studies with 7117 patients; grade ≥ 3AEs included 13 treatment groups from 34 studies, with 7034 patients. The P score and rank of each treatment for each outcome are shown in Table 2.

**Table 2:**
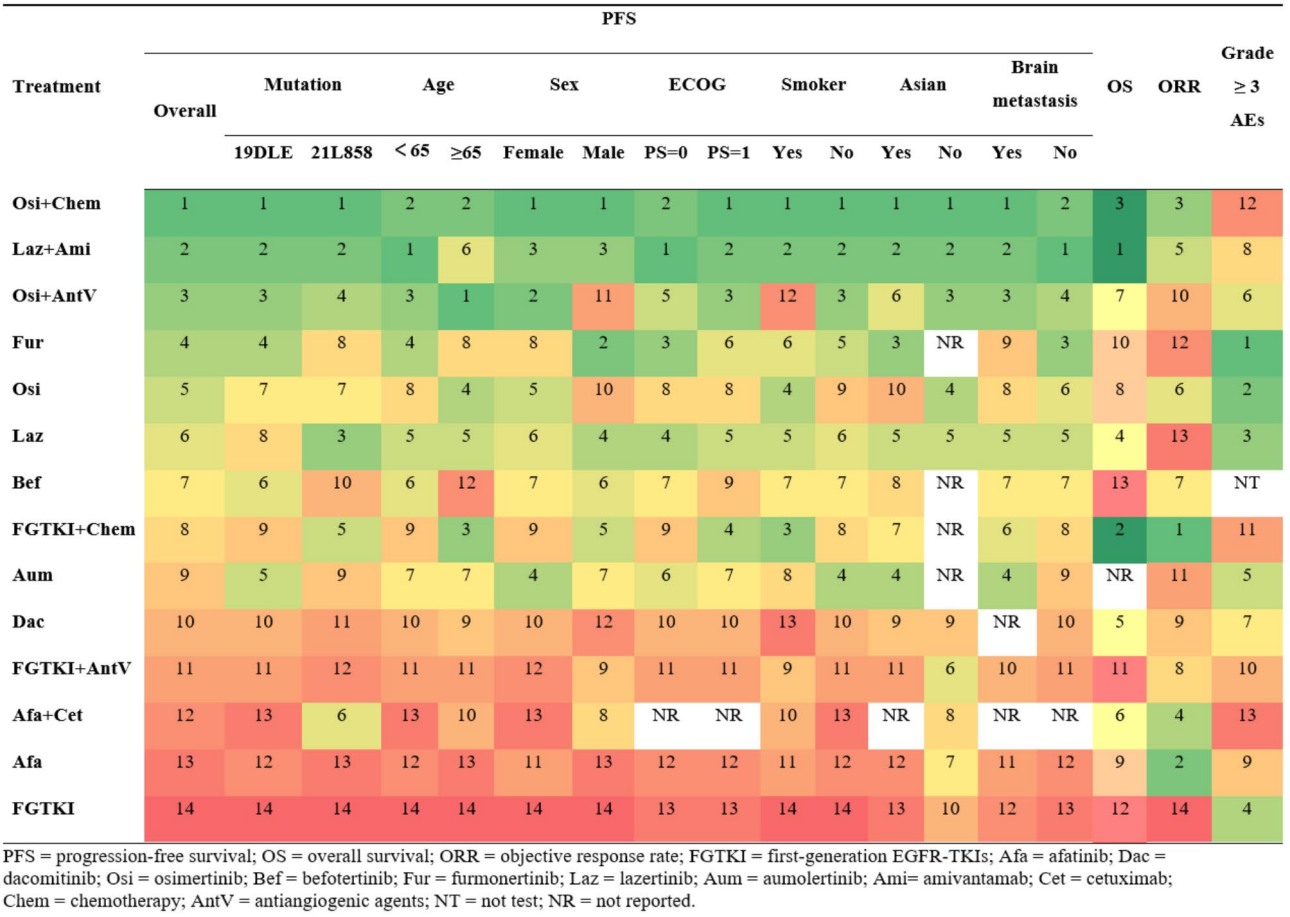
Results of P-score ranking profiles of each outcome

**Fig. 3:**
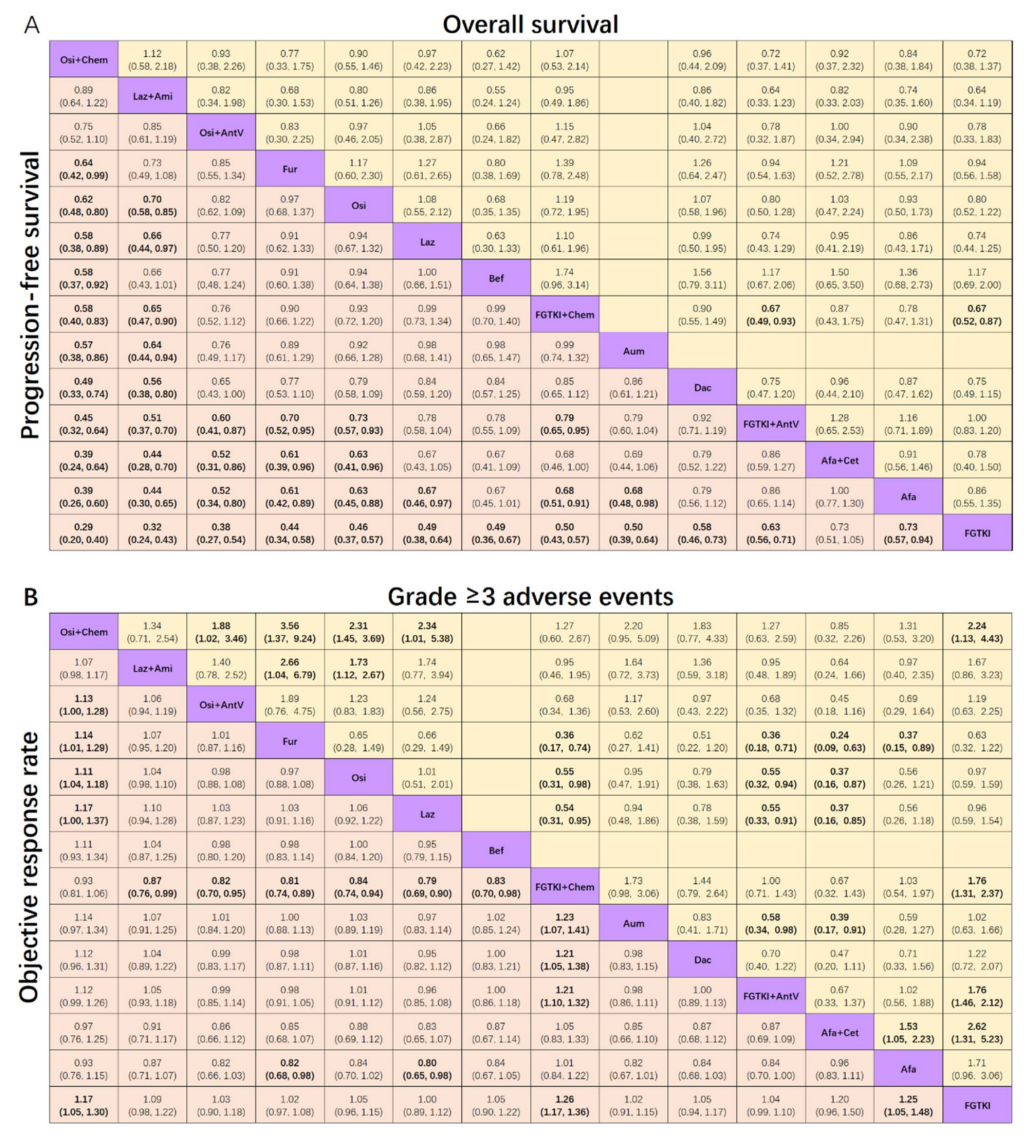
Pooled estimates of the network meta-analysis. **A**: Pooled hazard ratios (95% confidence intervals) for progression-free survival (lower triangle) and overall survival (upper triangle) in patients with advanced EGFR-mutated NSCLC. **B**: Pooled risk ratios (95% confidence intervals) for objective response rate (lower triangle) and grade ≥ 3 adverse events (upper triangle) in patients with advanced EGFR-mutated NSCLC. The data in each cell represent hazard or risk ratios (95% confidence intervals) comparing the treatment defined in the column with the treatment defined in the row. Significant results are indicated in bold

Regarding progression-free survival as shown in Fig. 3A, osimertinib plus chemotherapy and lazertinib plus amivantamab ranked first and second, both demonstrating superiority in significantly prolonging progression-free survival compared to other treatments, except for osimertinib plus antiangiogenic agents and two new third-generation EGFR-TKIs (Osi + Chem versus Osi + AntV (HR 0.75, 95% CI 0.52 to 1.10), Laz + Ami versus Osi + AntV (HR 0.85, 95% CI 0.61 to 1.19), Laz + Ami versus Fur (HR 0.73, 95% CI 0.49 to 1.08), Laz + Ami versus Bef (HR 0.66, 95% CI 0.43 to 1.01)). Furmonertinib ranked the highest among the five third-generation EGFR-TKIs. First-generation EGFR-TKIs plus chemotherapy exhibited efficacy comparable to third-generation EGFR-TKIs, while first-generation EGFR-TKIs alone showed the worst progression-free survival compared to other treatments.

Regarding overall survival as shown in Fig. 3A, lazertinib plus amivantamab ranked first, followed by first-generation EGFR-TKIs plus chemotherapy and osimertinib plus chemotherapy. First-generation EGFR-TKIs plus chemotherapy significantly prolonged overall survival compared to first-generation EGFR-TKIs plus anti-angiogenic agents (HR 0.67, 95% CI 0.49 to 0.93) and first-generation EGFR-TKIs (HR 0.67, 95% CI 0.52 to 0.87). However, no significant improvement in overall survival was observed with lazertinib plus amivantamab or osimertinib plus chemotherapy compared to the other treatment regimens.

Regarding the objective response rate as shown in Fig. 3B, first-generation EGFR-TKIs plus chemotherapy ranked first. It showed a significant difference compared to most other treatments, except for osimertinib plus chemotherapy (RR 0.93, 95% CI 0.81 to 1.06), afatinib (RR 1.01, 95% CI 0.84 to 1.22), and afatinib plus cetuximab (RR 1.05, 95% CI 0.83 to 1.33). Afatinib ranked second, while lazertinib plus amivantamab ranked third. Furthermore, befotertinib was likely the least effective treatment in achieving an objective response.

Regarding grade ≥ 3 AEs as shown in Fig. 3B, the lowest incidence of grade ≥ 3 AEs was observed with furmonertinib, while the highest incidence was noted with afatinib plus cetuximab. Adding treatments such as chemotherapy or antiangiogenic agents to an EGFR-TKI regimen was associated with a higher occurrence of grade ≥ 3 AEs (Osi + Chem versus Osi (RR 2.31, RR 1.45 to 3.69), FGTKI + Chem versus FGTKI (RR 1.76, RR 1.31 to 2.37), FGTKI + AntV versus FGTKI (RR 1.76, RR 1.46 to 2.12)).

18 of the most common specific AEs were also analyzed (eFigure 2), including any grade and grade ≥ 3 AEs. We found that adding other treatments to EGFR-TKIs would increase the AEs related to the additional treatment but did not increase the AEs related to the EGFR-TKIs (eFigure 3). Additional chemotherapy increased neutropenia (Osi + Chem versus Osi (RR 7.53, RR 3.83 to 14.79)) and anemia (Osi + Chem versus Osi (RR 5.80, RR 2.31 to 14.58)) (eFigure 3 J and 3 K). Additional antiangiogenic agents increased proteinuria (Osi + AntV versus Osi (RR 8.25, RR 3.11 to 21.87)), hemorrhage (FGTKI + AntV versus FGTKI (RR 2.51, RR 1.79 to 3.52)) and hypertension (FGTKI + AntV versus FGTKI (RR 3.68, RR 2.54 to 5.32)) (eFigure 3 M, 3 N and 3O). Additional amivantamab increased venous thrombotic events (Laz + Ami versus Laz (RR 46.43, RR 1.02 to 2117.90)) (eFigure 3P).

#### Subgroup analysis based on EGFR mutation types (exon 19 deletions and exon 21 L858R mutations)

The analysis of progression-free survival in patient subgroups with exon 19 deletions and the exon 21 L858R mutation included 14 treatment groups across 24 studies involving 4443 and 3104 patients, respectively. Among the treatments, osimertinib plus chemotherapy and lazertinib plus amivantamab ranked first and second, respectively, in efficacy.

**Fig. 4:**
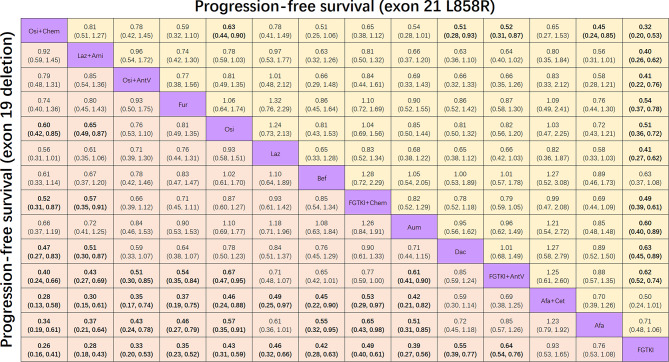
Pooled estimates for progression-free survival of subgroup analyses for patients with exon 19 deletion (lower triangle) and exon 21 L858R subgroups (upper triangle). The data in each cell represent hazard ratios (95% confidence intervals) comparing the treatment defined in the column with the treatment defined in the row. Significant results are indicated in bold

For patients with exon 19 deletions, as shown in Fig. 4, osimertinib plus chemotherapy (HR 0.60, 95% CI 0.42 to 0.85) and lazertinib plus amivantamab (HR 0.65, 95% CI 0.49 to 0.87) both provided longer progression-free survival compared to osimertinib alone, as well as to first and second-generation EGFR-TKIs and their combination treatments.

For patients with the exon 21 L858R mutation (Fig. 4), osimertinib plus chemotherapy significantly prolonged progression-free survival compared to osimertinib alone (HR 0.63, 95% CI 0.44 to 0.91), as well as compared to first and second-generation EGFR-TKIs and first-generation EGFR-TKIs plus antiangiogenic agents. Lazertinib plus amivantamab (HR 0.78, 95% CI 0.59 to 1.03) showed a trend towards more prolonged progression-free survival compared to osimertinib alone, though this difference did not reach statistical significance.

For both subgroups, osimertinib plus chemotherapy and lazertinib plus amivantamab did not demonstrate superior efficacy in prolonging progression-free survival compared to befotertinib, furmonertinib, lazertinib, and aumolertinib and osimertinib plus antiangiogenic agents.

#### Subgroup analysis based on various clinicopathological characteristics

**Fig. 5:**
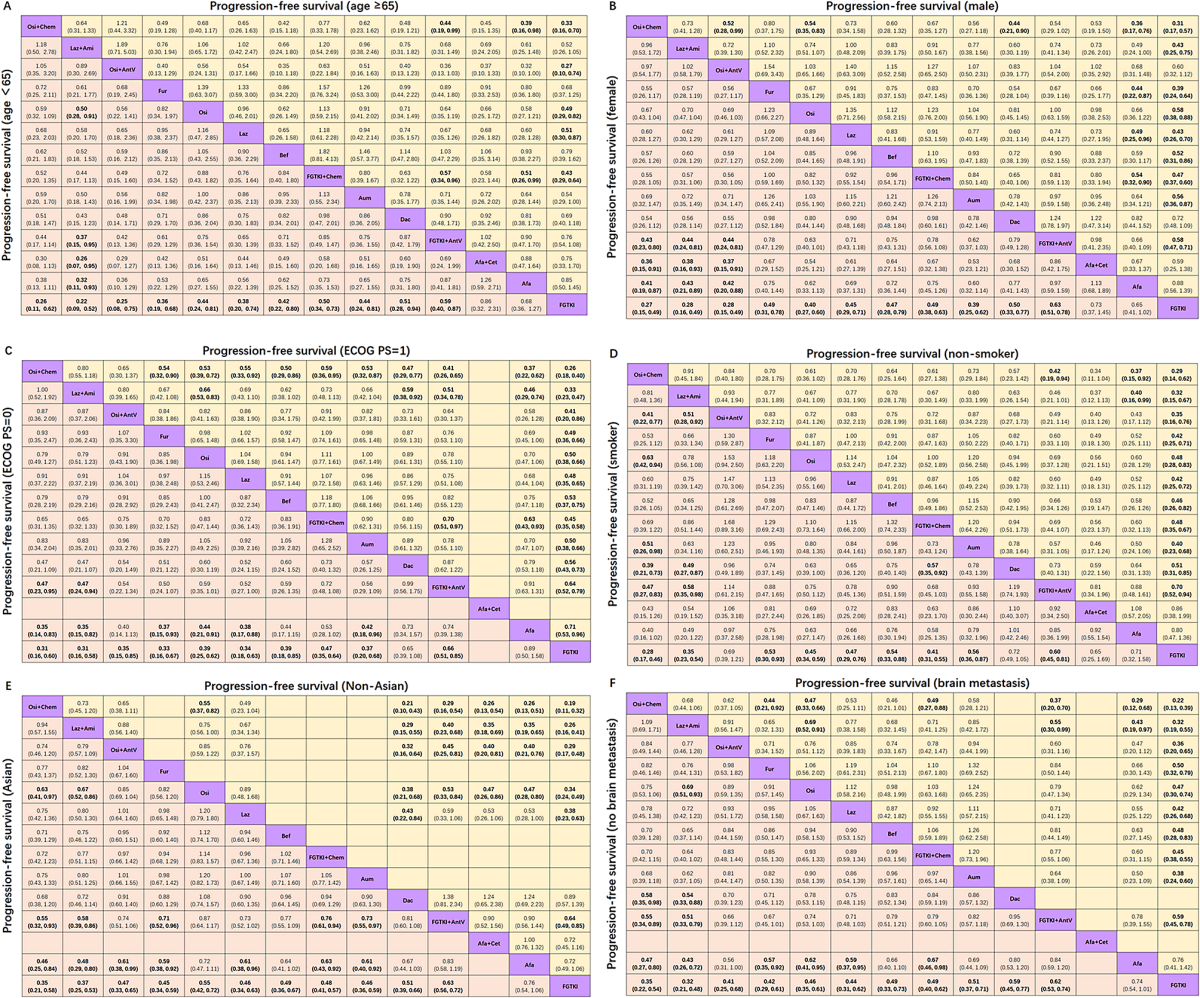
Pooled estimates for progression-free survival of subgroup analyses based on various clinicopathological characteristics. **A**-**F**: age < 65 (lower triangle) and age ≥ 65 years (upper triangle), female (lower triangle) and male (upper triangle), ECOG PS of 0 (lower triangle) and ECOG PS of 1(upper triangle), smoker (lower triangle) and non-smoker (upper triangle), Asian (lower triangle) and Non-Asian (upper triangle), no brain metastasis (lower triangle) and brain metastasis (upper triangle). The data in each cell represent hazard or risk ratios (95% confidence intervals) comparing the treatment defined in the column with the treatment defined in the row. Significant results are in bold

Subgroup analysis based on clinicopathological characteristics, including age, sex, Eastern Cooperative Oncology Group (ECOG) status, smoking status, ethnicity, and brain metastasis, showed that osimertinib plus chemotherapy and lazertinib plus amivantamab ranked first and second for almost all clinicopathological characteristics (Table 2). The details of the pooled estimates for progression-free survival of subgroup analysis based on various clinicopathological characteristics are presented in Fig. 5. Osimertinib plus chemotherapy provided more benefit than most other treatments for patients with ECOG PS = 1 (Fig. 5C). For smokers, osimertinib plus chemotherapy (HR 0.41, 95% CI 0.22 to 0.77) and lazertinib plus amivantamab (HR 0.51, 95% CI 0.28 to 0.92) significantly prolonged progression-free survival compared to osimertinib plus antiangiogenic agents (Fig. 5D). For patients with brain metastasis, osimertinib plus chemotherapy demonstrated a trend towards longer progression-free survival compared to lazertinib plus amivantamab (HR 0.68, 95% CI 0.44 to 1.06) (Fig. 5F). New Asian third-generation EGFR-TKIs did not significantly differ in prolonging progression-free survival compared to osimertinib for these clinicopathological characteristics. Additionally, osimertinib plus antiangiogenic agents did not differ substantially in prolonging progression-free survival compared to third-generation EGFR-TKIs (Fig. 5).

### Sensitivity analyses

We performed sensitivity analyses, which included 18 phase III trials, to ascertain the reliability and robustness of the primary results derived from the network meta-analysis. The outcomes of these sensitivity analyses were broadly consistent with the primary results (eFigure 4).

### Heterogeneity, inconsistency, and small-study effects

Consistency, heterogeneity, and publication bias are presented in Table 3. Our assessment revealed minimal heterogeneity across all comparisons. However, some inconsistency was observed for the objective response rate and serious adverse events, primarily from FGTKI versus FGTKI + Chem and FGTKI versus FGTKI + AntV. The net split function was not employed to evaluate consistency between direct and indirect evidence because no loop existed. A funnel plot was used to visually demonstrate small-study effects, revealing no publication bias for all outcomes (eFigure 5).

**Table 3:** Tests for inconsistency, heterogeneity, and small-study effects

Outcome	Heterogeneity	Consistency		Small-Study Effects
	τ^2	Q value	*P* value	Egger’s test *P* value
Progression-free survival	0	10.76	0.631	0.943
Overall survival	0.034	21.37	0.029	0.134
Objective response rate	0	8.08	0.838	0.423
Grade ≥ 3 adverse events	0.045	36.36	< 0.001	0.213

Heterogeneity across studies was quantified using the restricted maximum likelihood (REML) method. Inconsistency was evaluated using Cochran’s Q test. Small-study effects were assessed using Begg’s test. Inconsistencies are in red

## Discussion

In this systematic review and network meta-analysis, we identified 26 randomized controlled trials involving 8,359 patients and compared 14 treatment groups. This study goes beyond previous analyses by incorporating the most recent trials, including newer third-generation EGFR-TKIs and their combination treatments, which have not been thoroughly evaluated in prior meta-analyses [39, 51]. Additionally, we conducted subgroup analyses based on specific clinicopathological characteristics, such as EGFR mutation types, age, and smoking status, providing detailed insights into treatment efficacy across diverse patient populations. By comparing adverse event profiles alongside efficacy, our study offers a more comprehensive evaluation of EGFR-TKI monotherapies and combination strategies for advanced EGFR-mutated NSCLC. These findings contribute valuable guidance for clinical decision-making in selecting optimal first-line treatments for this patient group.

Firstly, our analysis indicates that osimertinib plus pemetrexed with platinum chemotherapy and lazertinib plus amivantamab demonstrate superior efficacy in progression-free survival for overall EGFR-mutated patients, potentially guiding treatment choices in this cohort. Secondly, adding antiangiogenic agents to osimertinib did not significantly improve progression-free survival. Third, new third-generation EGFR-TKIs exhibited high efficacy, showing similar progression-free survival outcomes compared to osimertinib alone. Finally, combination treatments generally led to increased toxicity, and the increased toxicity spectrums and events were mainly related to the additional agents.

EGFR-TKIs have been established as standard first-line treatment for patients with EGFR-mutated advanced NSCLC, a conclusion primarily drawn from the trial’s demonstration of superior progression-free and overall survival benefits compared to chemotherapy [15, 52–54]. Unfortunately, acquired resistance will inevitably occur in patients after receiving EGFR-TKIs, leading to tumor recurrence and metastasis. To prevent or delay acquired resistance to EGFR-TKIs and prolong overall survival, combining EGFR-TKIs with other anti-tumor therapies, such as antiangiogenic agents or chemotherapy, is a rational strategy [19]. 

NEJ026 was the first multicenter phase III trial to demonstrate that bevacizumab plus erlotinib combination therapy improves progression-free survival compared with erlotinib alone in patients with EGFR-positive NSCLC [12]. Subsequent phase III trials have demonstrated the superiority of combining first-generation EGFR-TKIs with antiangiogenic agents over monotherapy in improving progression-free survival for patients with advanced NSCLC harboring EGFR mutations [55–63]. The mechanism by which the combination of antiangiogenic drugs and EGFR-TKIs provides benefits may stem from the ability of antiangiogenic agents to normalize blood flow in tumor blood vessels, thereby enhancing drug delivery and concentration within tumors [64, 65]. Additionally, antiangiogenic agents might delay the emergence of resistance mechanisms [55]. Now, Erlotinib plus bevacizumab or ramucirumab have also received recommendations in international guidelines. We found that first-generation EGFR-TKIs plus antiangiogenic agents could improve progression-free survival and objective response rate than first-generation EGFR-TKIs. Despite these positive results, this combination strategy did not exhibit efficacy surpassing that of third-generation EGFR-TKIs and first-generation EGFR-TKIs plus chemotherapy.

First-generation EGFR-TKIs plus chemotherapy is another common combination strategy, and the efficacy was established by several phase II and III trials [38, 66–70]. Some authors hypothesize that the progression-free survival benefit may be attributed to simultaneous treatment preventing the emergence of gefitinib resistance mediated by the T790M mutation [39, 71]. While this hypothesis is supported by in vitro tests, clinical trial results indicate that the occurrence rate of the T790M mutation did not significantly differ between treatment groups [66, 69, 72]. Our NMA found that first-generation EGFR-TKIs plus chemotherapy ranked first for objective response rate and could significantly increase objective response rate than first-generation EGFR-TKIs (RR 1.26, 95% CI 1.17 to 1.36) and showed better efficacy in prolonging progression-free survival than first-generation EGFR-TKIs plus antiangiogenic agents (HR 0.79, 95% CI 0.64 to 0.96), but did not provide longer progression-free survival than third-generation EGFR-TKIs. These results are consistent with the findings reported by previous studies [66, 67]. We also found some results that differed from earlier reports; specifically, our results show that first-generation EGFR-TKIs plus chemotherapy significantly prolonged overall survival compared to EGFR-TKIs plus antiangiogenic agents (HR 0.67, 95% CI 0.49 to 0.93). Though the second progression-free survival was not included in our analysis, first-generation EGFR-TKIs plus chemotherapy showed significant improvements compared to first-generation EGFR-TKIs alone [67, 68, 72]. The progression-free survival and overall survival benefits suggest that these combination treatments present a promising alternative, particularly for patients without access to third-generation EGFR-TKIs.

Though osimertinib is the preferred first-line option for advanced EGFR-mutated NSCLC, its high cost previously hindered its cost-effectiveness as a first-line treatment in many countries [73–77]. Developing new EGFR-TKIs, including third- and fourth-generation EGFR-TKIs, is one way to address these issues [22, 78–80]. To date, a total of five third-generation EGFR-TKIs have been approved by various food and drug administrations, including osimertinib, befotertinib, furmonertinib, lazertinib, and aumolertinib. Except for osimertinib, the four new TKIs were developed by Asian countries [23–25, 81]. No study has compared the efficacy of these TKIs among patients with advanced EGFR-mutated NSCLC. In our NMA, although these TKIs show better efficacy than first-generation EGFR-TKIs, none of the new third-generation EGFR-TKIs surpassed the effectiveness of osimertinib. Their efficacy and safety profiles did not show significant differences. Lazertinib conducted phase III trials involving both Asian and non-Asian patients; the phase III trials for befotertinib, furmonertinib, and aumolertinib only involved Chinese patients [23, 25, 81]. Consequently, confirming the efficacy of these Chinese third-generation EGFR-TKIs in a broader, non-Chinese patient population necessitates further clinical trials.

Building on the successful results of first-generation EGFR-TKIs combined with antiangiogenic agents, a few phase II trials have evaluated the efficacy of osimertinib plus antiangiogenic agents for previously untreated patients with advanced NSCLC harboring EGFR mutations [82–84]. In a pooled analysis of these trials, we did not find better efficacy of the combination treatments compared to osimertinib alone. WJOG9717L was the first trial to report the results of EGFR-TKIs combined with Bevacizumab, with a negative outcome [82]. The authors thought that the negative results may be influenced by the short duration of exposure to bevacizumab. Two other trials, OSIRAM-1 and RAMOSE [83, 84], evaluated osimertinib combined with ramucirumab, as reported at ESMO 2023. In the RAMOSE trial, which had a positive result, the median duration of treatment with ramucirumab was 14.2 months. However, in the OSIRAM-1 trial, which had a negative result, the median duration of treatment was only 4.7 months. Although there were differences in randomization ratios, follow-up schedules, ramucirumab usage, stratification factors, and primary endpoints between the two trials, the results suggested that a longer exposure time to antiangiogenic agents’ treatment may be key to achieving positive outcomes. However, this hypothesis requires further, well-designed trials to be established.

Recent evidence shows that adding chemotherapy to osimertinib extended the benefits of osimertinib alone. In the FLAURA2 [21], first-line treatment with osimertinib plus chemotherapy led to significantly longer progression-free survival compared to osimertinib monotherapy in patients with EGFR-mutated advanced NSCLC (HR, 0.62; 95% CI 0.49 to 0.79). This result suggests that the combination overcomes intratumor heterogeneity by eliciting an additive effect by targeting different cell populations, thereby improving clinical outcomes. In our NMA, osimertinib plus chemotherapy ranked first in prolonging progression-free survival and is the only treatment showing superior efficacy compared to all third-generation EGFR-TKIs. Although osimertinib plus chemotherapy did not show superior efficacy in extending overall survival, this may be due to the interim analysis being still immature (data maturity, 27%). However, second progression-free survival (HR, 0.70; 95% CI 0.52 to 0.93), a surrogate endpoint for overall survival, indicated that initial treatment with osimertinib plus chemotherapy provides better efficacy for subsequent therapy. Now, osimertinib plus chemotherapy has been approved by the FDA and recommended by NCCN guidelines [85]. As follow-up time extends, if the final overall survival is prolonged by osimertinib plus chemotherapy, the FLAURA2 strategy will be considered a superior standard first-line therapy for EGFR-mutated advanced NSCLC.

MET amplification has been identified as a primary mechanism of acquired resistance to third-generation EGFR-TKI treatment, including osimertinib [86–88]. Amivantamab, a bispecific monoclonal antibody targeting EGFR and MET, has unique mechanisms of action, including ligand blocking, receptor degradation, and engagement of immune effector cells through its optimized Fc domain [89–91]. The MARIPOSA trial revealed a 30% reduction in the risk of disease progression or death for patients treated with lazertinib plus amivantamab compared to those receiving osimertinib monotherapy (HR, 0.70; 95% CI 0.58 to 0.85), with respective progression-free survival of 23.7 months and 16.6 months [20]. Amivantamab has also shown promising antitumor efficacy in combination with chemotherapy as a first-line treatment for patients with advanced NSCLC harboring EGFR exon 20 insertions [92]. It notably improved progression-free survival compared to chemotherapy alone (6.3 versus 4.2 months, respectively) in patients who had progressed after osimertinib [93]. In our NMA, lazertinib plus amivantamab ranked second and is one of the only two treatments that prolonged progression-free survival compared to third-generation EGFR-TKIs. Like osimertinib plus chemotherapy, we also found that lazertinib plus amivantamab did not provide overall survival than osimertinib alone. We also did not find a significant difference between lazertinib plus amivantamab and osimertinib plus chemotherapy in terms of survival efficacy.

Some existing evidence has proven that the efficacy of TKIs varies across EGFR mutation subtypes and patients’ clinicopathological characteristics [39, 51]. In our NMA, subgroup analyses showed some differences. Lazertinib plus amivantamab didn’t prolong progression-free survival among patients over 65 years compared to osimertinib. However, when age stratification was set at 75 years, lazertinib plus amivantamab demonstrated superior efficacy in both subgroups, although the authors did not explain these results [20]. Befotertinib and furmonertinib also did not prove superior efficacy among patients over 65 years compared to first-generation EGFR-TKIs for prolonging progression-free survival. Befotertinib also did not prove superior efficacy among patients with the exon 21 L858R mutation. Except for these differences, the efficacy of osimertinib plus chemotherapy, lazertinib plus amivantamab, and third-generation EGFR-TKIs showed consistency in subgroup analyses compared to overall groups.

EGFR-TKI monotherapy is associated with less toxicity, with the most common adverse events being rash, stomatitis, paronychia, dry skin, and constipation. Combination strategies increase the risk of additional adverse events due to the added drugs. Chemotherapy is associated with hematotoxicity, antiangiogenic agents with proteinuria, hemorrhage, hypertension, and amivantamab with venous thrombotic events and infusion-related reactions. Clinicians should balance the toxicity and survival benefits when prescribing combination therapy.

In our NMA, we found that afatinib plus cetuximab had the worst safety profile without progression-free survival improvement, and furmonertinib had the best safety profile. A previous study showed that icotinib had the best safety profile [39]. Although the toxicity of befotertinib is comparable to other TKIs, icotinib has the lowest toxicity. Since only one phase III trial included a comparison between befotertinib and icotinib [25], including befotertinib in the grade ≥ 3 adverse events analysis would relatively increase its perceived toxicity compared to other TKIs. Thus, befotertinib was not pooled in our analysis for grade ≥ 3 adverse events in our NMA. In the PALOMA-3 trial [94], subcutaneous amivantamab significantly decreased infusion-related reactions and venous thrombotic events while prolonging progression-free survival and overall survival. Third-generation EGFR-TKI plus subcutaneous amivantamab may be the best choice for advanced EGFR-mutated NSCLC.

Moreover, we found differences in toxicity spectrums and occurrence rates among EGFR-TKIs. Afatinib has the highest occurrence rate of rash, diarrhea, and stomatitis, while dacomitinib has the highest occurrence rate of paronychia. Osimertinib has higher TKI-related toxicity events compared to other third-generation EGFR-TKIs. To optimize management and treatment selection, understanding the key adverse events of each EGFR-TKI over long-term use is essential.

### Limitations

Our study has several limitations. Firstly, we merged multiple first-generation EGFR-TKI monotherapies into one treatment group. This approach prevents the comparison of each first-generation EGFR-TKI monotherapy with other regimens. Moreover, the drug mechanisms are not identical among first-generation TKIs, and merging them may introduce bias in the NMA. Secondly, some trial results were based on interim results, and the absence of overall survival data in some trials limits the robustness of our analysis. Thirdly, including randomized controlled trials from conference abstracts, which often lack complete information on clinical characteristics, poses challenges for conducting comprehensive analyses. Fourthly, some inconsistencies in overall survival and adverse events existed in our NMA, which could affect the reliability of our analyses. Fifthly, there were fewer randomized controlled trials for some treatments (such as new third-generation EGFR-TKIs), particularly in subgroup analyses, which could impact the reliability of our findings. Finally, questions regarding the efficacy of treatments in sequential or maintenance use were not investigated and, therefore, remain a subject for further studies.

## Conclusion

Our network meta-analysis shows that osimertinib plus chemotherapy and lazertinib plus amivantamab are superior first-line treatment options for patients with advanced EGFR-mutated NSCLC. However, it is important to note that combination treatments were associated with a higher incidence of adverse events. Therefore, cautious application and vigilant monitoring in clinical practice are necessary.

## Electronic supplementary material

Below is the link to the electronic supplementary material.

Supplementary Material 1

## Data Availability

The data of this study can be obtained from the corresponding author according to reasonable requirements.
