# Correction: EGFR-TKIs or EGFR-TKIs combination treatments for untreated advanced EGFR-mutated NSCLC: a network meta-analysis

**DOI:** 10.1186/s12885-025-13577-3

**Published:** 2025-01-28

**Authors:** Ao Liu, Xiaoming Wang, Lian Wang, Han Zhuang, Liubo Xiong, Xiao Gan, Qian Wang, Guanyu Tao

**Affiliations:** Department of Respiratory Medicine, Chengdu BOE Hospital, Chengdu, Sichuan Province 610000 China

**Correction: BMC Cancer 24, 1390 (2024)**

10.1186/s12885-024-13168-8

Following publication of the original article [1], It was reported that the efficacy and safety of various first-line EGFR-TKI monotherapies and combination treatments for advanced EGFR-mutated non-small cell lung cancer (NSCLC) were assessed. An error occurred during the statistical analysis of the FURLONG study (Furmonertinib vs. first-generation EGFR-TKI) in the Overall Survival (OS) data. Specifically, the HR value for OS in the FURLONG study was incorrectly recorded during the statistical analysis. The previous (incorrect) HR value was HR 0.41, 95% CI 0.31 to 0.55. The corrected value is HR 0.94, 95% CI 0.65 to 1.36. This mistake led to errors in the OS analysis for targeted therapy. As a result, the following sections of the manuscript have been affected by this error: OS League Table (Figure 3 (including the sensitivity analysis for the OS League Table, eFigure 4 in supplementary material 1)), P-SCORE ranking profiles (Table 2), Heterogeneity analysis (Table 3), OS funnel plot (eFigure 5), and Egger’s test (eFigure 5). The updated supplementary material 1 is given in this correction article (which includes eFigure 4 and eFigure 5).

The HR values for overall survival (upper triangle in Figure 3A) comparing Furmonertinib to other treatments are incorrect. The other HR values are correct.

**Incorrect Figure 3**

**Fig. 3:**
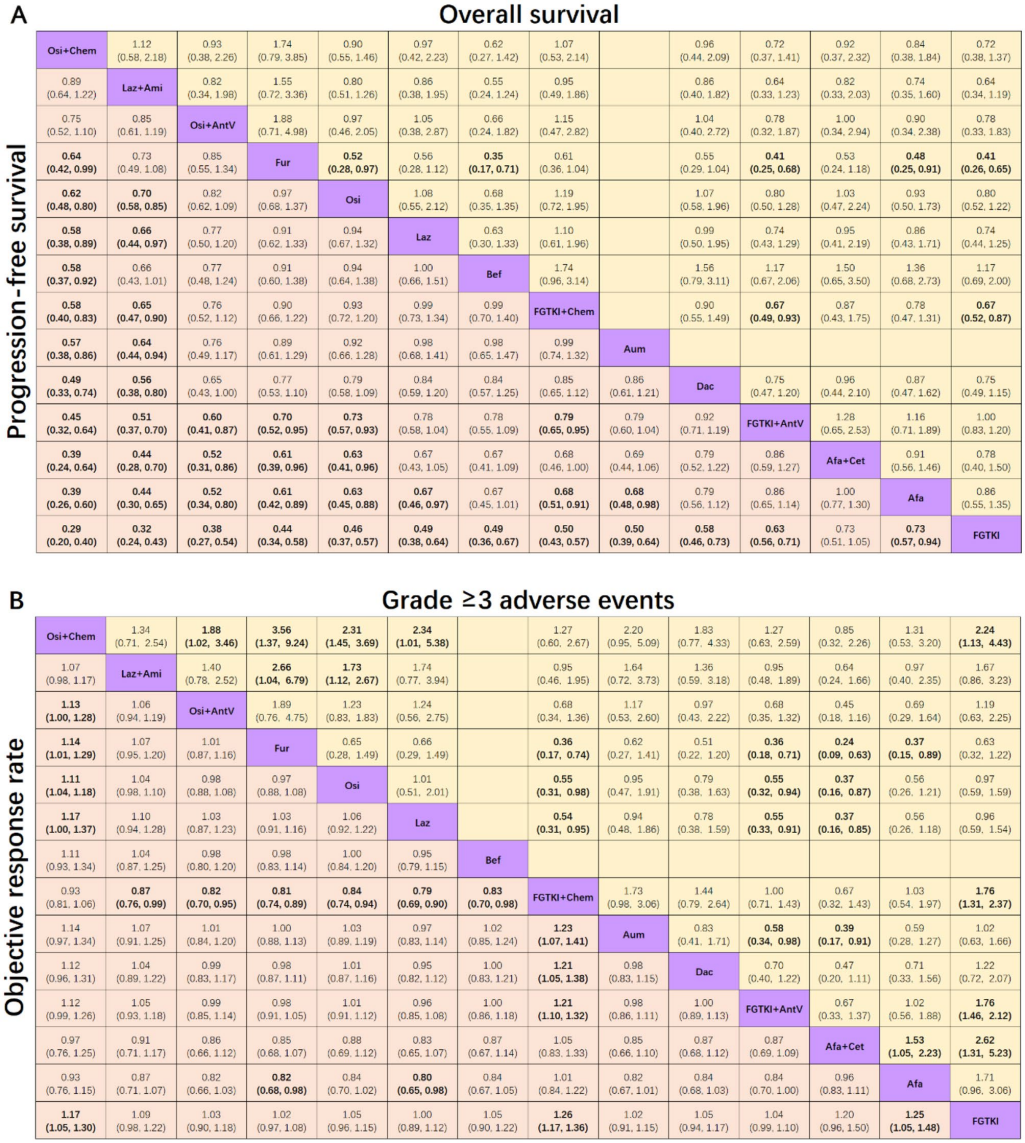
Pooled estimates of the network meta-analysis. **A**: Pooled hazard ratios (95% confidence intervals) for progression-free survival (lower triangle) and overall survival (upper triangle) in patients with advanced EGFR-mutated NSCLC. **B**: Pooled risk ratios (95% confidence intervals) for objective response rate (lower triangle) and grade ≥ 3 adverse events (upper triangle) in patients with advanced EGFR-mutated NSCLC. The data in each cell represent hazard or risk ratios (95% confidence intervals) comparing the treatment defined in the column with the treatment defined in the row. Significant results are indicated in bold

**Corrected Figure 3**

**Fig. 3:**
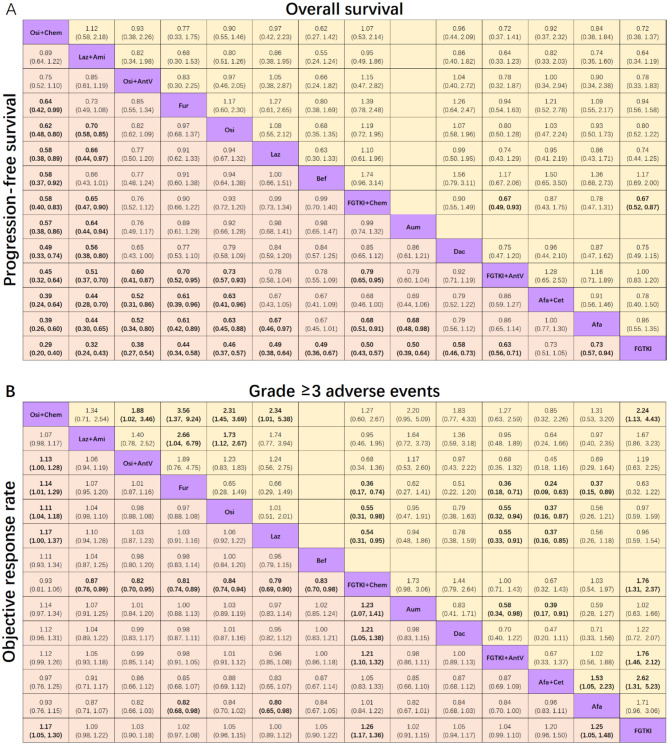
Pooled estimates of the network meta-analysis. **A**: Pooled hazard ratios (95% confidence intervals) for progression-free survival (lower triangle) and overall survival (upper triangle) in patients with advanced EGFR-mutated NSCLC. **B**: Pooled risk ratios (95% confidence intervals) for objective response rate (lower triangle) and grade ≥ 3 adverse events (upper triangle) in patients with advanced EGFR-mutated NSCLC. The data in each cell represent hazard or risk ratios (95% confidence intervals) comparing the treatment defined in the column with the treatment defined in the row. Significant results are indicated in bold

The P-score ranking profiles for OS are incorrect; the other rankings are correct.

**Incorrect Table 2**

**Table 2:**
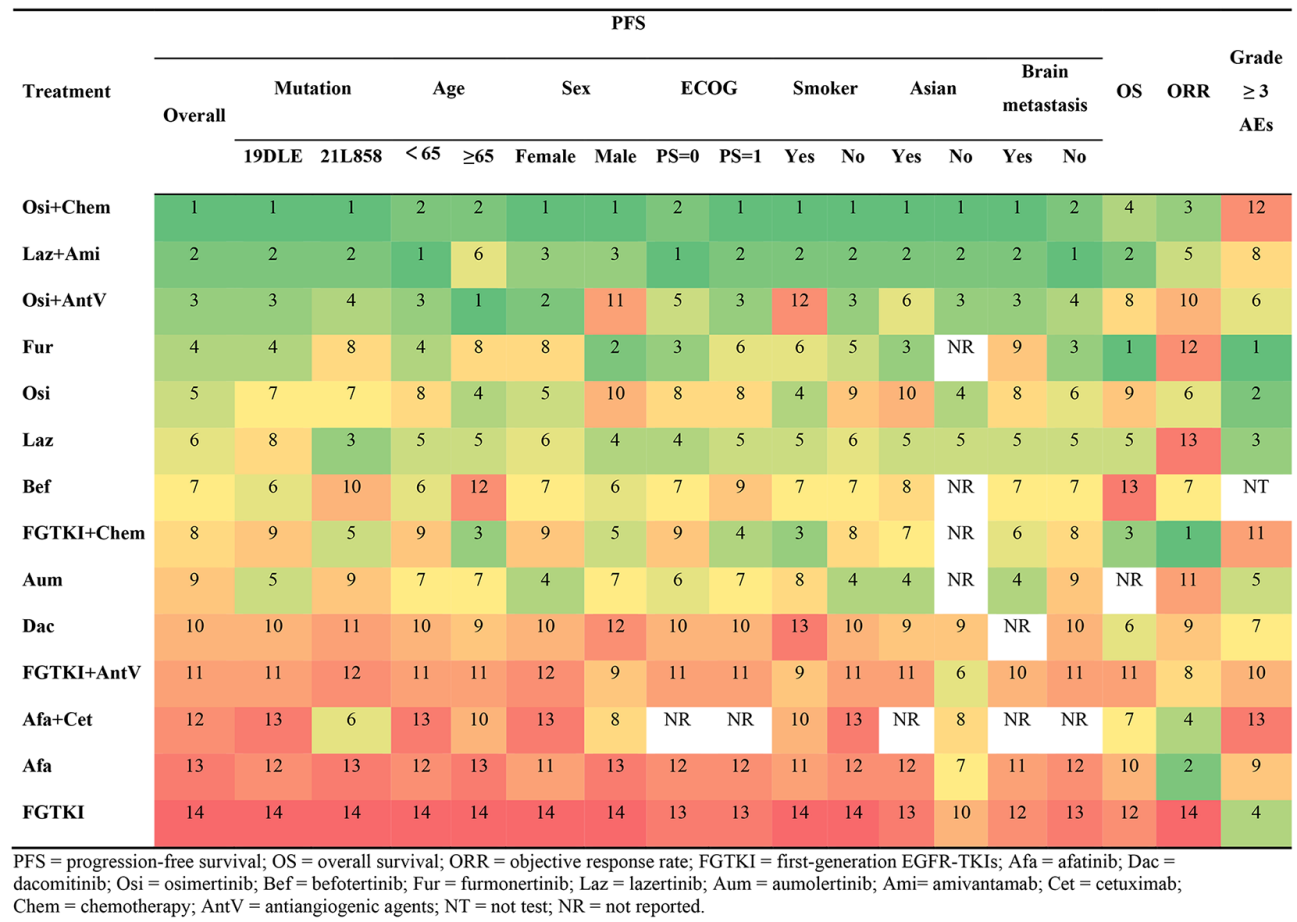
Results of P-score ranking profiles of each outcome

**Corrected Table 2**

**Table 2:**
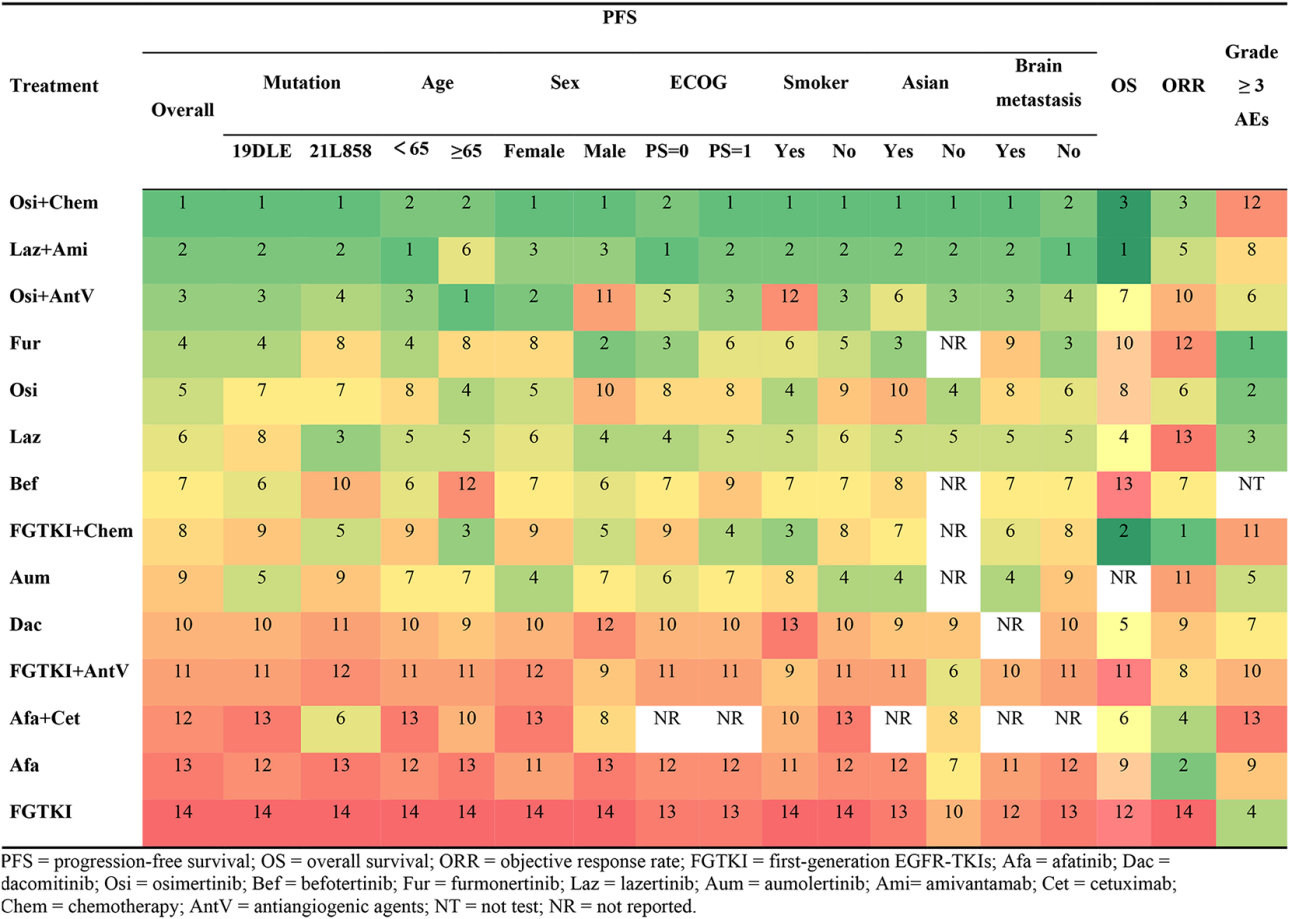
Results of P-score ranking profiles of each outcome

The Egger’s test P-value for overall survival is incorrect; the other values are correct.

**Incorrect Table 3**

**Table 3:** Tests for inconsistency, heterogeneity, and small-study effects

Outcome	Heterogeneity		Consistency			Small-Study Effects
	τ^2		Q value	*P* value		Egger’s test *P* value
Progression-free survival	0		10.76	0.631		0.943
Overall survival	0.034		21.37	0.029		0.139
Objective response rate	0		8.08	0.838		0.423
Grade ≥ 3 adverse events	0.045		36.36	< 0.001		0.213

Heterogeneity across studies was quantified using the restricted maximum likelihood (REML) method. Inconsistency was evaluated using Cochran’s Q test. Small-study effects were assessed using Egger’s test. Inconsistencies are in red

**Corrected Table 3**

**Table 3:** Tests for inconsistency, heterogeneity, and small-study effects

Outcome	Heterogeneity		Consistency			Small-Study Effects
	τ^2		Q value	*P* value		Egger’s test *P* value
Progression-free survival	0		10.76	0.631		0.943
Overall survival	0.034		21.37	0.029		0.134
Objective response rate	0		8.08	0.838		0.423
Grade ≥ 3 adverse events	0.045		36.36	< 0.001		0.213

Heterogeneity across studies was quantified using the restricted maximum likelihood (REML) method. Inconsistency was evaluated using Cochran’s Q test. Small-study effects were assessed using Egger’s test. Inconsistencies are in red

The HR values for overall survival (upper triangle in eFigure 4 A) comparing Furmonertinib to other treatments are incorrect. The other HR values are correct. The corrected figure can be found in supplementary material 1.

The P-value in eFigure 5B is incorrect. The corrected figure can be found in supplementary material 1.

Further to this, the authors have noticed some errors in the following section:

**The results section about overall survival**

**(Incorrect)** Regarding overall survival as shown in Figure 3A, furmonertinib ranked first and significantly prolonged overall survival compared to osimertinib (HR 0.52, 95% CI 0.28 to 0.97), befotertinib (HR 0.35, 95% CI 0.17 to 0.71), first-generation EGFR-TKIs plus anti-angiogenic agents (HR 0.41, 95% CI 0.25 to 0.68), afatinib (HR 0.48, 95% CI 0.25 to 0.91), and first-generation EGFR-TKIs (HR 0.41, 95% CI 0.26 to 0.65). Lazertinib plus amivantamab and first-generation EGFR-TKIs plus chemotherapy ranked second and third, respectively. First-generation EGFR-TKIs plus chemotherapy significantly prolonged overall survival compared to first-generation EGFR-TKIs plus anti-angiogenic agents and first-generation EGFR-TKIs alone.

**(corrected)** Regarding overall survival as shown in Figure 3A, lazertinib plus amivantamab ranked first, followed by first-generation EGFR-TKIs plus chemotherapy and osimertinib plus chemotherapy. First-generation EGFR-TKIs plus chemotherapy significantly prolonged overall survival compared to first-generation EGFR-TKIs plus anti-angiogenic agents (HR 0.67, 95% CI 0.49 to 0.93) and first-generation EGFR-TKIs (HR 0.67, 95% CI 0.52 to 0.87). However, no significant improvement in overall survival was observed with lazertinib plus amivantamab or osimertinib plus chemotherapy compared to the other treatment regimens.

**The discussion section**

**(Incorrect)** In our NMA, we found that afatinib plus cetuximab had the worst safety profile without progression-free survival improvement, and furmonertinib had the best safety profile and longer overall survival compared to first-generation EGFR-TKIs, osimertinib, and befotertinib. This suggests that adverse events can affect survival, as they can lead to the discontinuation of treatments.

**(corrected)** In our NMA, we found that afatinib plus cetuximab had the worst safety profile without progression-free survival improvement, and furmonertinib had the best safety profile.

The original article [1] has been corrected.

## Electronic supplementary material

Below is the link to the electronic supplementary material.

Supplementary Material 1
