# Supplementary material for: EGFR-TKIs or EGFR-TKIs combination treatments for untreated advanced EGFR-mutated NSCLC: a network meta-analysis
Source: BMC Cancer. 2024 Nov 12;24:1390. doi: 10.1186/s12885-024-13168-8 (PMC11555867; doi:10.1186/s12885-024-13168-8)
Supplement: Supplementary file 1 — Supplementary Material 1 [file 12885_2024_13168_MOESM1_ESM.docx]

**Supplementary file**

**eTable 1.** Literature search strategy.

**eTable 2.** Checklist of the PRISMA extension for network meta-analysis.

**eFigure 1.** Quality Assessment using the revised tool for risk of bias in randomized trials.

**eFigure 2.** A frequency toxicity profile showing the percentage incidence of each specific adverse event based on the treatment population included.

**eFigure 3.** Pooled estimates of each specific adverse event.

**eFigure 4.** Sensitivity analysis including only phase III trials.

**eFigure5.** Funnel plots of the network meta-analysis.

**eTable 1.** Literature search strategy.

| **Search results of Pubmed** | | |
| --- | --- | --- |
| #1 | ("Carcinoma, Non-Small-Cell Lung"[Mesh] OR "nsclc"[Title/Abstract]) OR (("lung cancer*"[Title/Abstract] OR "lung carcinom*"[Title/Abstract] OR "lung neoplasm*"[Title/Abstract] OR "lung tumo*"[Title/Abstract]) AND ("non small cell*"[Title/Abstract] OR "nonsmall cell*"[Title/Abstract])) | 109,539 |
| #2 | (("EGFR"[Title/Abstract] OR "epidermal growth factor receptor"[Title/Abstract]) AND ("TKI"[Title/Abstract] OR "tyrosine kinase inhibitor"[Title/Abstract])) OR "Gefitinib"[Title/Abstract] OR "Iressa"[Title/Abstract] OR "Erlotinib"[Title/Abstract] OR "Tarceva"[Title/Abstract] OR "Dacomitinib"[Title/Abstract] OR "Vizimpro"[Title/Abstract] OR "Icotinib"[Title/Abstract] OR "Conmana"[Title/Abstract] OR "Afatinib"[Title/Abstract] OR "Gilotrif"[Title/Abstract] OR "Osimertinib"[Title/Abstract] OR "Tagrisso"[Title/Abstract] OR "Aumolertinib"[Title/Abstract] OR "almonertinib"[Title/Abstract] OR "Furmonertinib"[Title/Abstract] OR "Befotertinib"[Title/Abstract] OR "lazertinib"[Title/Abstract] OR "Leclaza"[Title/Abstract] | 21,556 |
| #3 | (randomized controlled trial[pt] OR controlled clinical trial[pt] OR randomized[tiab] OR placebo[tiab] OR clinical trials as topic[mesh:noexp] OR randomly[tiab] OR trial[ti]) NOT (animals [mh] NOT (humans [mh] AND animals[mh])) | 1,498,514 |
| #4 | #1 AND #2 AND #3 | 1,759 |
| **Search results of Emabse** | | |
| #1 | 'carcinoma, non-small-cell lung'/exp OR 'nsclc':ti,ab,kw OR (('lung cancer*':ti,ab,kw OR 'lung carcinom*':ti,ab,kw OR 'lung neoplasm*':ti,ab,kw OR 'lung tumo*':ti,ab,kw) AND ('non small cell*':ti,ab,kw OR 'nonsmall cell*':ti,ab,kw)) | 249,760 |
| #2 | ('egfr':ti,ab,kw OR 'epidermal growth factor receptor':ti,ab,kw) AND ('tki':ti,ab,kw OR 'tyrosine kinase inhibitor':ti,ab,kw) OR 'gefitinib':ti,ab,kw OR 'iressa':ti,ab,kw OR 'erlotinib':ti,ab,kw OR 'tarceva':ti,ab,kw OR 'dacomitinib':ti,ab,kw OR 'vizimpro':ti,ab,kw OR 'icotinib':ti,ab,kw OR 'conmana':ti,ab,kw OR 'afatinib':ti,ab,kw OR 'gilotrif':ti,ab,kw OR 'osimertinib':ti,ab,kw OR 'tagrisso':ti,ab,kw OR 'aumolertinib':ti,ab,kw OR 'almonertinib':ti,ab,kw OR 'furmonertinib':ti,ab,kw OR 'befotertinib':ti,ab,kw OR 'lazertinib':ti,ab,kw OR 'leclaza':ti,ab,kw | 38,147 |
| #3 | 'crossover procedure':de OR 'double-blind procedure':de OR 'randomized controlled trial':de OR 'single-blind procedure':de OR random*:de,ab,ti OR factorial*:de,ab,ti OR crossover*:de,ab,ti OR ((cross NEXT/1 over*):de,ab,ti) OR placebo*:de,ab,ti OR ((doubl* NEAR/1 blind*):de,ab,ti) OR ((singl* NEAR/1 blind*):de,ab,ti) OR assign*:de,ab,ti OR allocat*:de,ab,ti OR volunteer*:de,ab,ti | 3,364,213 |
| #4 | #1 AND #2 AND #3 | 4075 |
| **Search results of CENTRAL** | | |
| #1 | MeSH descriptor: [Carcinoma, Non-Small-Cell Lung] explode all trees | 6655 |
| #2 | (nsclc):ti,ab,kw | 12202 |
| #3 | (lung cancer*):ti,ab,kw | 31975 |
| #4 | (lung carcinom*):ti,ab,kw | 11776 |
| #5 | (lung neoplasm*):ti,ab,kw | 16333 |
| #6 | (lung tumo*):ti,ab,kw | 12971 |
| #7 | #3 OR #4 OR #5 OR #6 | 35620 |
| #8 | (non small cell*):ti,ab,kw | 19503 |
| #9 | (nonsmall cell*):ti,ab,kw | 11280 |
| #10 | #8 OR #9 | 19561 |
| #11 | #7 AND #10 | 17266 |
| #12 | #1 OR #2 OR #11 | 18037 |
| #13 | ("EGFR" OR "epidermal growth factor receptor"):ti,ab,kw | 16988 |
| #14 | ("TKI" OR "tyrosine kinase inhibitor"):ti,ab,kw | 3602 |
| #15 | #13 AND #14 | 1466 |
| #16 | ("Gefitinib" OR "Iressa" OR "Erlotinib" OR "Tarceva" OR "Dacomitinib" OR "Vizimpro" OR "Icotinib" OR "Conmana" OR "Afatinib" OR "Gilotrif" OR "Osimertinib" OR "Tagrisso" OR "Aumolertinib" OR "almonertinib" OR "Furmonertinib" OR "Befotertinib" OR "lazertinib" OR "Leclaza"):ti,ab,kw | 3483 |
| #17 | #15 OR #16 | 4009 |
| #18 | #12 AND #17 | 2857 |

**eTable 2.** Checklist of the PRISMA extension for network meta-analysis.

| **Section/Topic** | **Item #** | **Checklist Item** | **Reported on Page #** |
| --- | --- | --- | --- |
| **TITLE** |  |  |  |
| Title | 1 | Identify the report as a systematic review *incorporating a network meta-analysis (or related form of meta-analysis).* | ***1*** |
|  |  |  |  |
| **ABSTRACT** |  |  |  |
| Structured summary | 2 | Provide a structured summary including, as applicable:  **Background:** main objectives  **Methods:** data sources; study eligibility criteria, participants, and interventions; study appraisal; and *synthesis methods, such as network meta-analysis.*  **Results:** number of studies and participants identified; summary estimates with corresponding confidence/credible intervals; *treatment rankings may also be discussed. Authors may choose to summarize pairwise comparisons against a chosen treatment included in their analyses for brevity.*  **Discussion/Conclusions:** limitations; conclusions and implications of findings.  **Other:** primary source of funding; systematic review registration number with registry name. | 3 |
|  |  |  |  |
| **INTRODUCTION** |  |  |  |
| Rationale | 3 | Describe the rationale for the review in the context of what is already known*, including mention of why a network meta-analysis has been conducted.* | ***4*** |
| Objectives | 4 | Provide an explicit statement of questions being addressed, with reference to participants, interventions, comparisons, outcomes, and study design (PICOS). | 4-5 |
|  |  |  |  |
| **METHODS** |  |  |  |
| Protocol and registration | 5 | Indicate whether a review protocol exists and if and where it can be accessed (e.g., Web address); and, if available, provide registration information, including registration number. | 5 |
| Eligibility criteria | 6 | Specify study characteristics (e.g., PICOS, length of follow-up) and report characteristics (e.g., years considered, language, publication status) used as criteria for eligibility, giving rationale. *Clearly describe eligible treatments included in the treatment network, and note whether any have been clustered or merged into the same node (with justification).* | ***5-6*** |
| Information sources | 7 | Describe all information sources (e.g., databases with dates of coverage, contact with study authors to identify additional studies) in the search and date last searched. | 5 |
| Search | 8 | Present full electronic search strategy for at least one database, including any limits used, such that it could be repeated. | 5 |
| Study selection | 9 | State the process for selecting studies (i.e., screening, eligibility, included in systematic review, and, if applicable, included in the meta-analysis). | 6 |
| Data collection process | 10 | Describe method of data extraction from reports (e.g., piloted forms, independently, in duplicate) and any processes for obtaining and confirming data from investigators. | 6 |
| Data items | 11 | List and define all variables for which data were sought (e.g., PICOS, funding sources) and any assumptions and simplifications made. | - |
| **Geometry of the network** | **S1** | Describe methods used to explore the geometry of the treatment network under study and potential biases related to it. This should include how the evidence base has been graphically summarized for presentation, and what characteristics were compiled and used to describe the evidence base to readers. |  |
| Risk of bias within individual studies | 12 | Describe methods used for assessing risk of bias of individual studies (including specification of whether this was done at the study or outcome level), and how this information is to be used in any data synthesis. | 6 |
| Summary measures | 13 | State the principal summary measures (e.g., risk ratio, difference in means). *Also describe the use of additional summary measures assessed, such as treatment rankings and surface under the cumulative ranking curve (SUCRA) values, as well as modified approaches used to present summary findings from meta-analyses.* | 7 |
| Planned methods of analysis | 14 | Describe the methods of handling data and combining results of studies for each network meta-analysis. This should include, but not be limited to:   - *Handling of multi-arm trials;* - *Selection of variance structure;* - *Selection of prior distributions in Bayesian analyses; and* - *Assessment of model fit.* | 7 |
| **Assessment of Inconsistency** | **S2** | Describe the statistical methods used to evaluate the agreement of direct and indirect evidence in the treatment network(s) studied. Describe efforts taken to address its presence when found. |  |
| Risk of bias across studies | 15 | Specify any assessment of risk of bias that may affect the cumulative evidence (e.g., publication bias, selective reporting within studies). | **7** |
| Additional analyses | 16 | Describe methods of additional analyses if done, indicating which were pre-specified. This may include, but not be limited to, the following:   - Sensitivity or subgroup analyses; - Meta-regression analyses; - *Alternative formulations of the treatment network; and* - *Use of alternative prior distributions for Bayesian analyses (if applicable).* | ***7*** |
|  |  |  |  |
| **RESULTS†** |  |  |  |
| Study selection | 17 | Give numbers of studies screened, assessed for eligibility, and included in the review, with reasons for exclusions at each stage, ideally with a flow diagram. | 8 |
| **Presentation of network structure** | **S3** | Provide a network graph of the included studies to enable visualization of the geometry of the treatment network. | ***8*** |
| **Summary of network geometry** | **S4** | Provide a brief overview of characteristics of the treatment network. This may include commentary on the abundance of trials and randomized patients for the different interventions and pairwise comparisons in the network, gaps of evidence in the treatment network, and potential biases reflected by the network structure. | ***8*** |
| Study characteristics | 18 | For each study, present characteristics for which data were extracted (e.g., study size, PICOS, follow-up period) and provide the citations. | 8 |
| Risk of bias within studies | 19 | Present data on risk of bias of each study and, if available, any outcome level assessment. | 8 |
| Results of individual studies | 20 | For all outcomes considered (benefits or harms), present, for each study: 1) simple summary data for each intervention group, and 2) effect estimates and confidence intervals. *Modified approaches may be needed to deal with information from larger networks.* | ***8-11*** |
| Synthesis of results | 21 | Present results of each meta-analysis done, including confidence/credible intervals. *In larger networks, authors may focus on comparisons versus a particular comparator (e.g. placebo or standard care), with full findings presented in an appendix. League tables and forest plots may be considered to summarize pairwise comparisons.* If additional summary measures were explored (such as treatment rankings), these should also be presented. | ***8-11*** |
| **Exploration for inconsistency** | **S5** | Describe results from investigations of inconsistency. This may include such information as measures of model fit to compare consistency and inconsistency models, *P* values from statistical tests, or summary of inconsistency estimates from different parts of the treatment network. | ***12*** |
| Risk of bias across studies | 22 | Present results of any assessment of risk of bias across studies for the evidence base being studied. | 12 |
| Results of additional analyses | 23 | Give results of additional analyses, if done (e.g., sensitivity or subgroup analyses, meta-regression analyses*, alternative network geometries studied, alternative choice of prior distributions for Bayesian analyses,* and so forth). | ***11*** |
|  |  |  |  |
| **DISCUSSION** |  |  |  |
| Summary of evidence | 24 | Summarize the main findings, including the strength of evidence for each main outcome; consider their relevance to key groups (e.g., healthcare providers, users, and policy-makers). | 12-18 |
| Limitations | 25 | Discuss limitations at study and outcome level (e.g., risk of bias), and at review level (e.g., incomplete retrieval of identified research, reporting bias). *Comment on the validity of the assumptions, such as transitivity and consistency. Comment on any concerns regarding network geometry (e.g., avoidance of certain comparisons).* | 18-19 |
| Conclusions | 26 | Provide a general interpretation of the results in the context of other evidence, and implications for future research. | 19 |
|  |  |  |  |
| **FUNDING** |  |  |  |
| Funding | 27 | Describe sources of funding for the systematic review and other support (e.g., supply of data); role of funders for the systematic review. This should also include information regarding whether funding has been received from manufacturers of treatments in the network and/or whether some of the authors are content experts with professional conflicts of interest that could affect use of treatments in the network. | ***2*** |

**eFigure 1.** Quality Assessment using the revised tool for risk of bias in randomized trials.

**eFigure 2.** A frequency toxicity profile showing the percentage incidence of each specific adverse event based on the treatment population included.

**
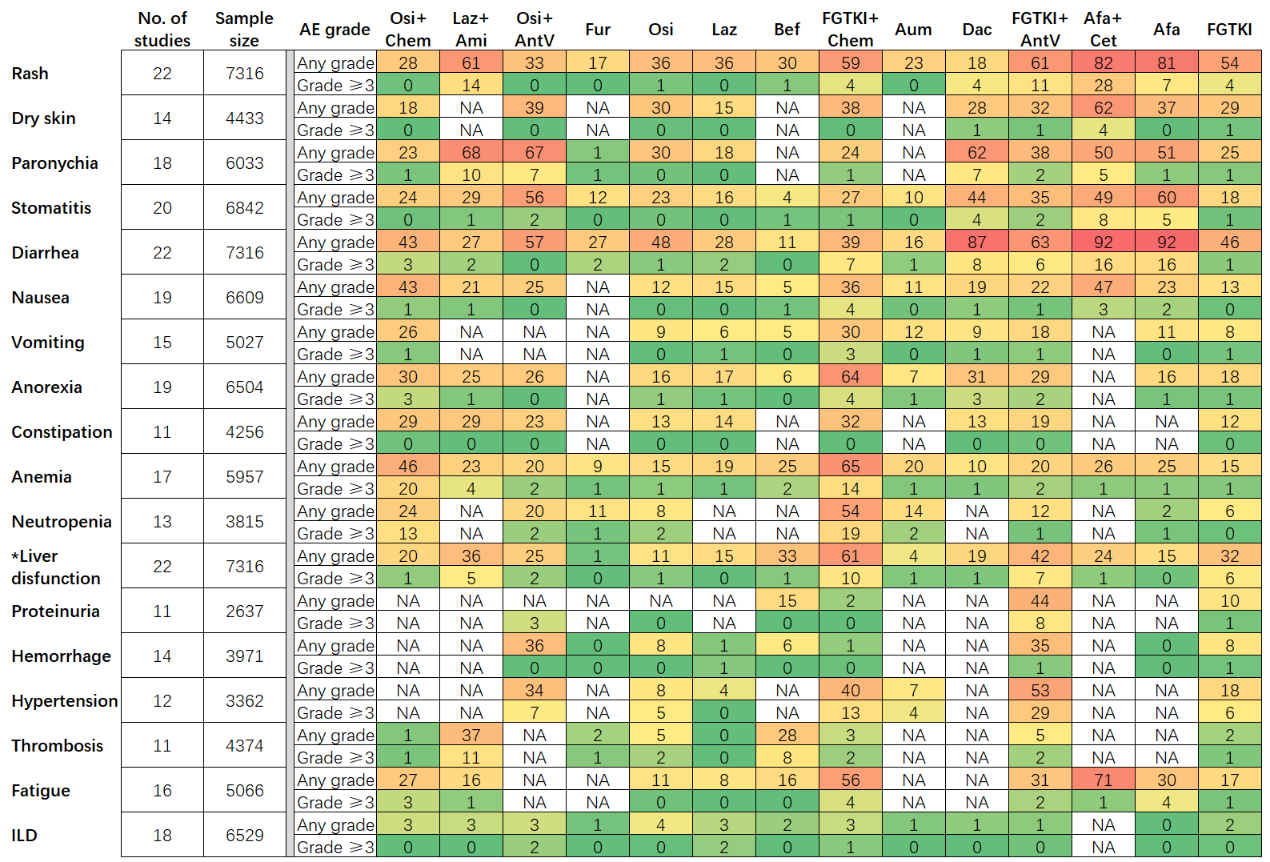
**

**Legend:** NA=not applicable; FGTKI = first-generation EGFR-TKIs; Afa = afatinib; Dac = dacomitinib; Osi = osimertinib; Bef = befotertinib; Fur = furmonertinib; Laz = lazertinib; Aum = aumolertinib; Ami= amivantamab; Cet = cetuximab; Chem = chemotherapy; AntV = antiangiogenic agents; ILD= interstitial lung disease. *When not reported, liver dysfunction was represented by alanine transaminase increased as it was reported in most studies.

**eFigure 3.** Pooled estimates of each specific adverse event.

**
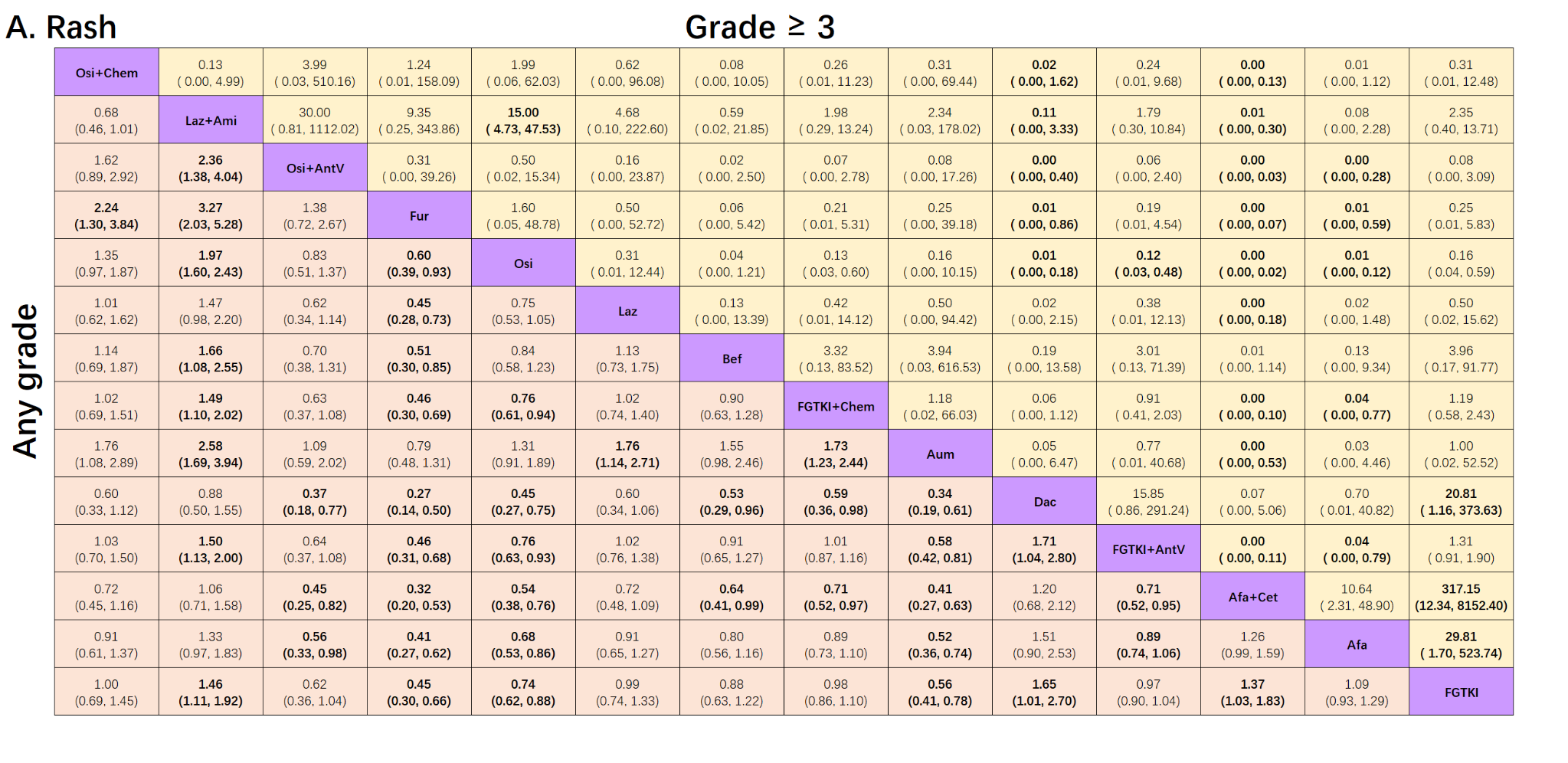
**

**Legend:** The lower triangle represents risk ratios for any grade of rash, while the upper triangle represents risk ratios for grade ≥ 3 rash. The data in each cell represent risk ratios (95% confidence intervals) comparing the treatment defined in the column with the treatment defined in the row. Significant results are indicated in bold. FGTKI = first-generation EGFR-TKIs; Afa = afatinib; Dac = dacomitinib; Osi = osimertinib; Bef = befotertinib; Fur = furmonertinib; Laz = lazertinib; Aum = aumolertinib; Ami= amivantamab; Cet = cetuximab; Chem = chemotherapy; AntV = antiangiogenic agents.

**
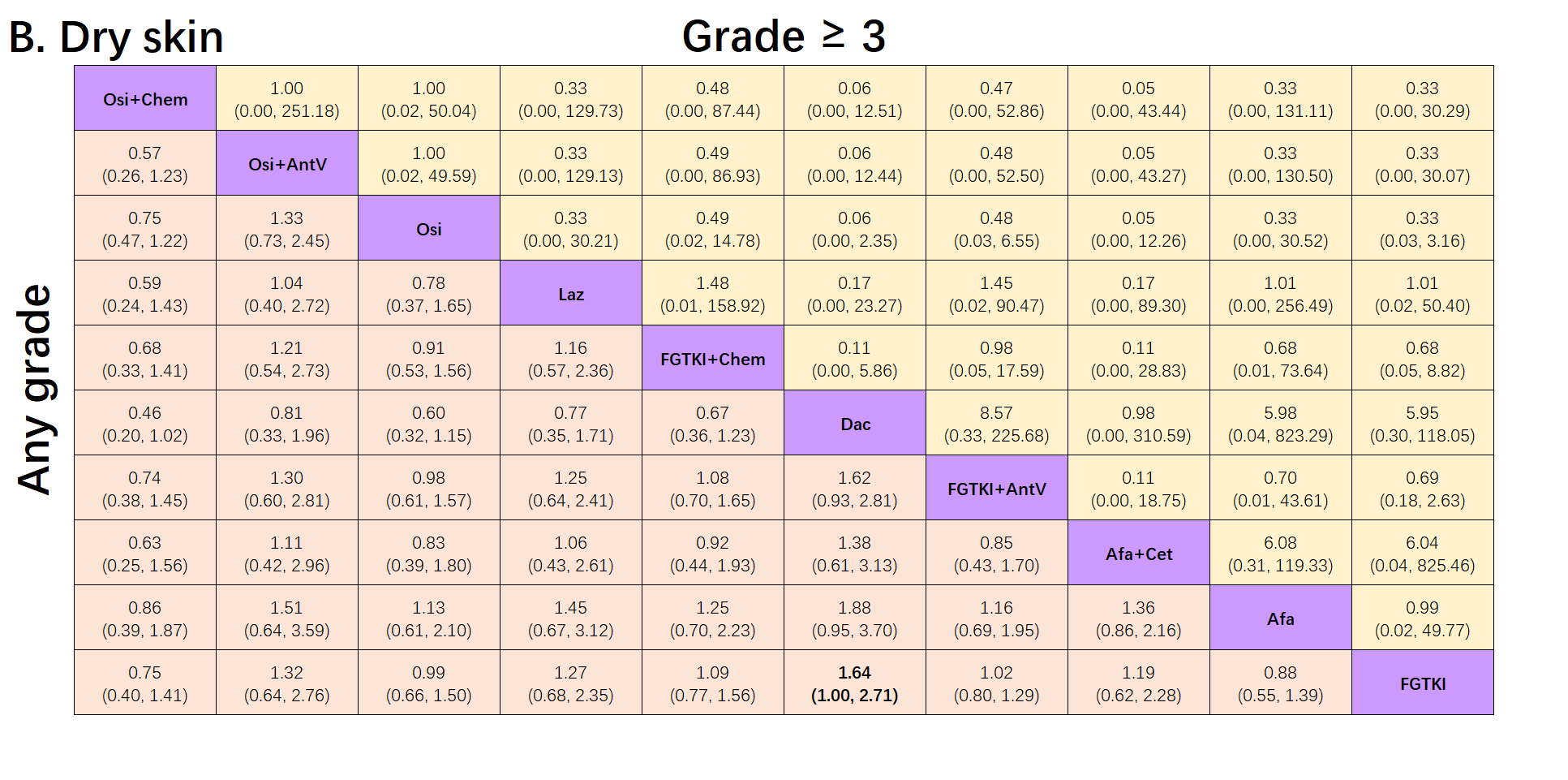
**

**Legend:** The lower triangle represents risk ratios for any grade of dry skin, while the upper triangle represents risk ratios for grade ≥ 3 dry skin. The data in each cell represent risk ratios (95% confidence intervals) comparing the treatment defined in the column with the treatment defined in the row. Significant results are indicated in bold. FGTKI = first-generation EGFR-TKIs; Afa = afatinib; Dac = dacomitinib; Osi = osimertinib; Laz = lazertinib; Cet = cetuximab; Chem = chemotherapy; AntV = antiangiogenic agents.

**
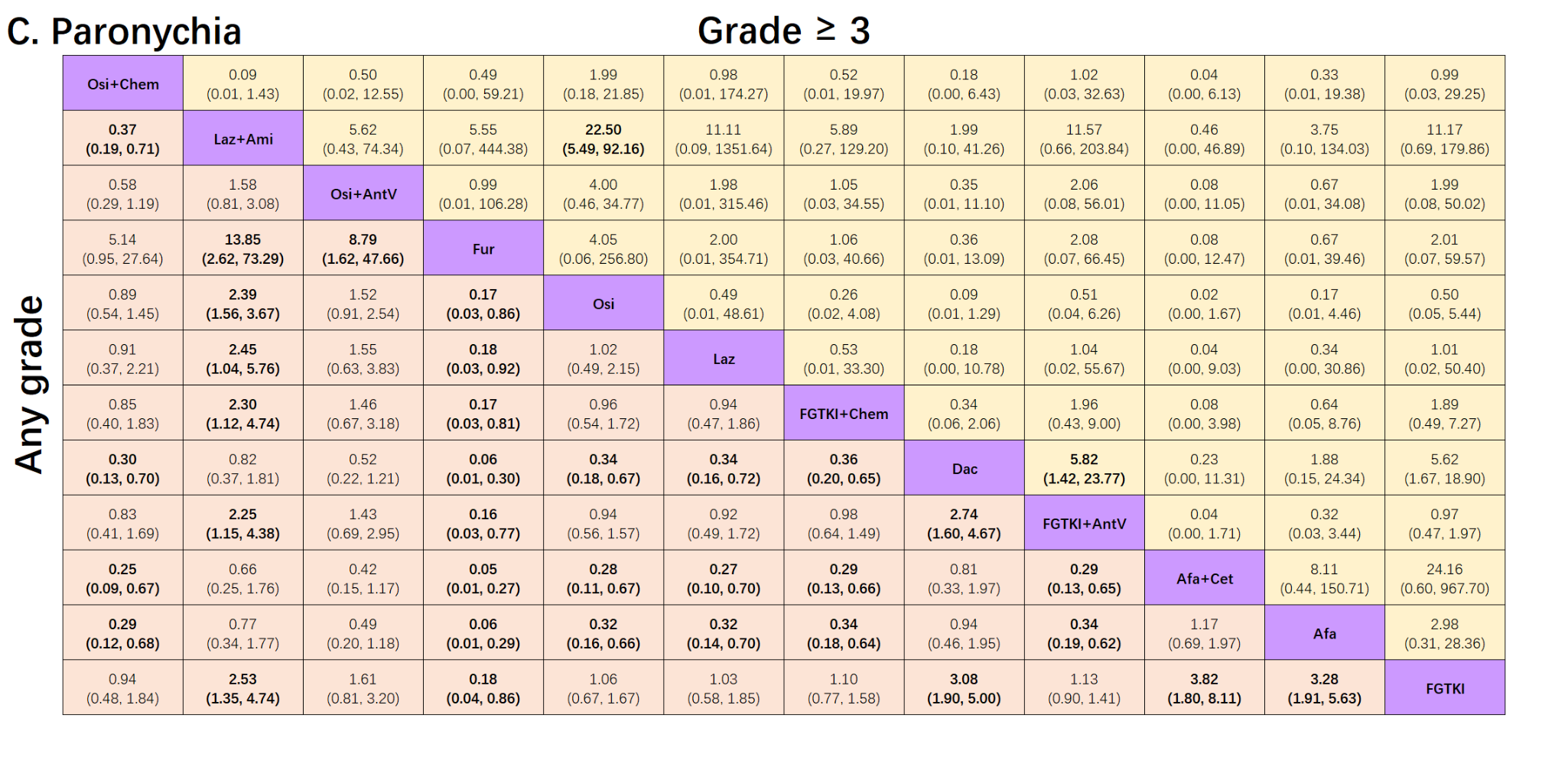
**

**Legend:** The lower triangle represents risk ratios for any grade of paronychia, while the upper triangle represents risk ratios for grade ≥ 3 paronychia. The data in each cell represent risk ratios (95% confidence intervals) comparing the treatment defined in the column with the treatment defined in the row. Significant results are indicated in bold. FGTKI = first-generation EGFR-TKIs; Afa = afatinib; Dac = dacomitinib; Osi = osimertinib; Fur = furmonertinib; Laz = lazertinib; Aum = aumolertinib; Ami= amivantamab; Cet = cetuximab; Chem = chemotherapy; AntV = antiangiogenic agents.

**
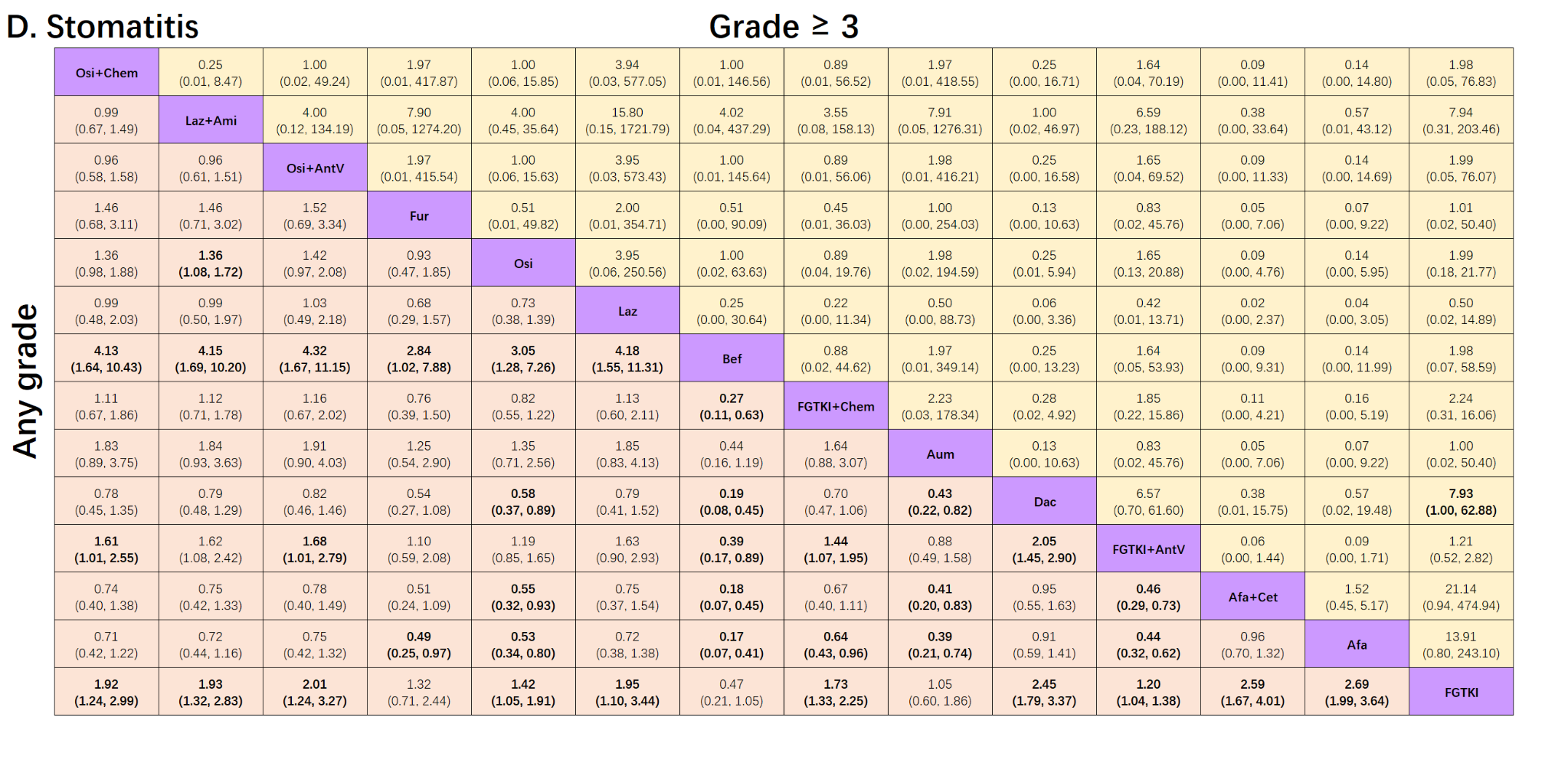
**

**Legend:** The lower triangle represents risk ratios for any grade of stomatitis, while the upper triangle represents risk ratios for grade ≥ 3 stomatitis. The data in each cell represent risk ratios (95% confidence intervals) comparing the treatment defined in the column with the treatment defined in the row. Significant results are indicated in bold. FGTKI = first-generation EGFR-TKIs; Afa = afatinib; Dac = dacomitinib; Osi = osimertinib; Bef = befotertinib; Fur = furmonertinib; Laz = lazertinib; Aum = aumolertinib; Ami= amivantamab; Cet = cetuximab; Chem = chemotherapy; AntV = antiangiogenic agents.

**
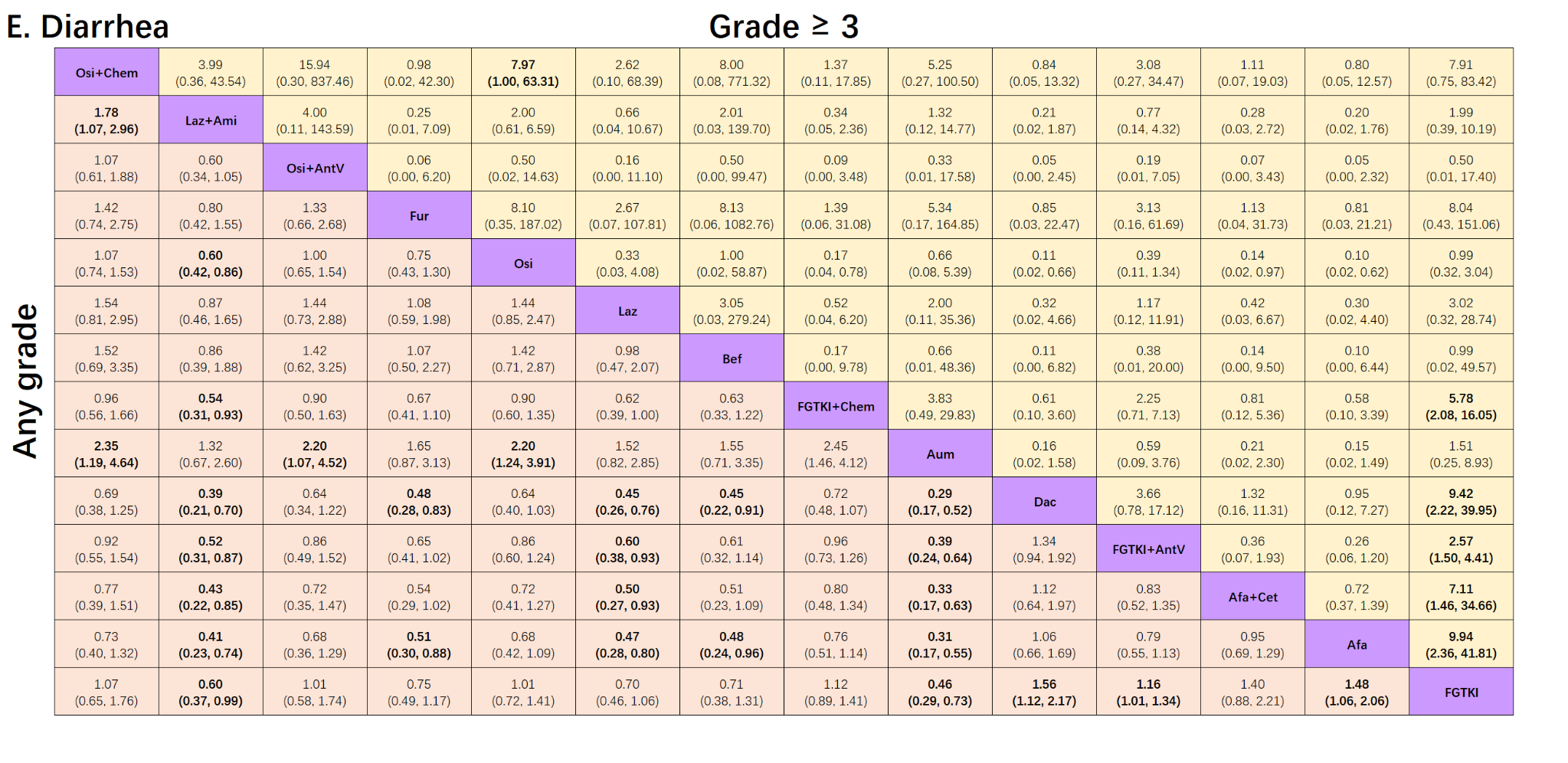
**

**Legend:** The lower triangle represents risk ratios for any grade of diarrhea, while the upper triangle represents risk ratios for grade ≥ 3 diarrhea. The data in each cell represent risk ratios (95% confidence intervals) comparing the treatment defined in the column with the treatment defined in the row. Significant results are indicated in bold. FGTKI = first-generation EGFR-TKIs; Afa = afatinib; Dac = dacomitinib; Osi = osimertinib; Bef = befotertinib; Fur = furmonertinib; Laz = lazertinib; Aum = aumolertinib; Ami= amivantamab; Cet = cetuximab; Chem = chemotherapy; AntV = antiangiogenic agents.

**
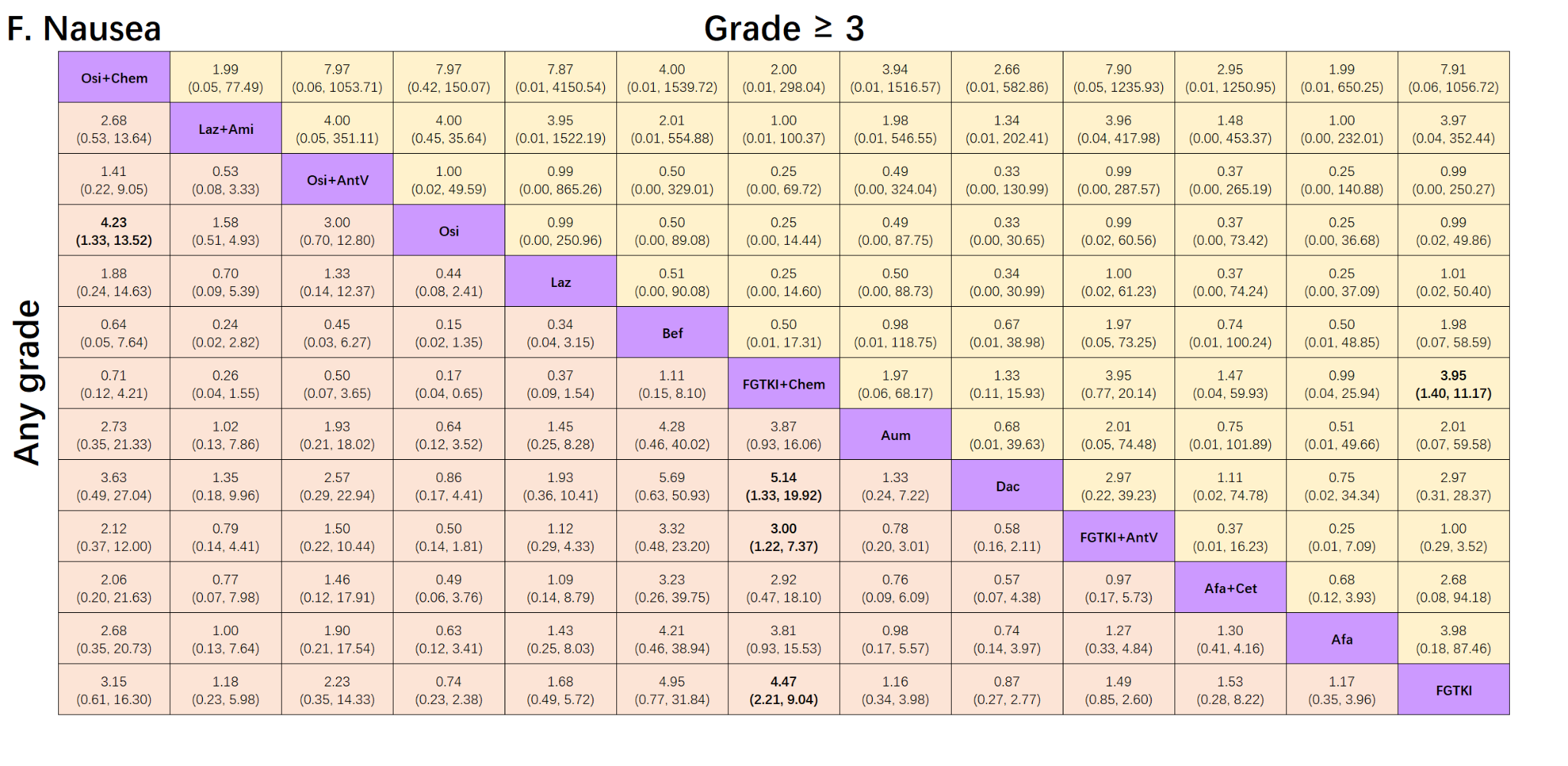
**

**Legend:** The lower triangle represents risk ratios for any grade of nausea, while the upper triangle represents risk ratios for grade ≥ 3 nausea. The data in each cell represent risk ratios (95% confidence intervals) comparing the treatment defined in the column with the treatment defined in the row. Significant results are indicated in bold. FGTKI = first-generation EGFR-TKIs; Afa = afatinib; Dac = dacomitinib; Osi = osimertinib; Bef = befotertinib; Laz = lazertinib; Aum = aumolertinib; Ami= amivantamab; Cet = cetuximab; Chem = chemotherapy; AntV = antiangiogenic agents.

**
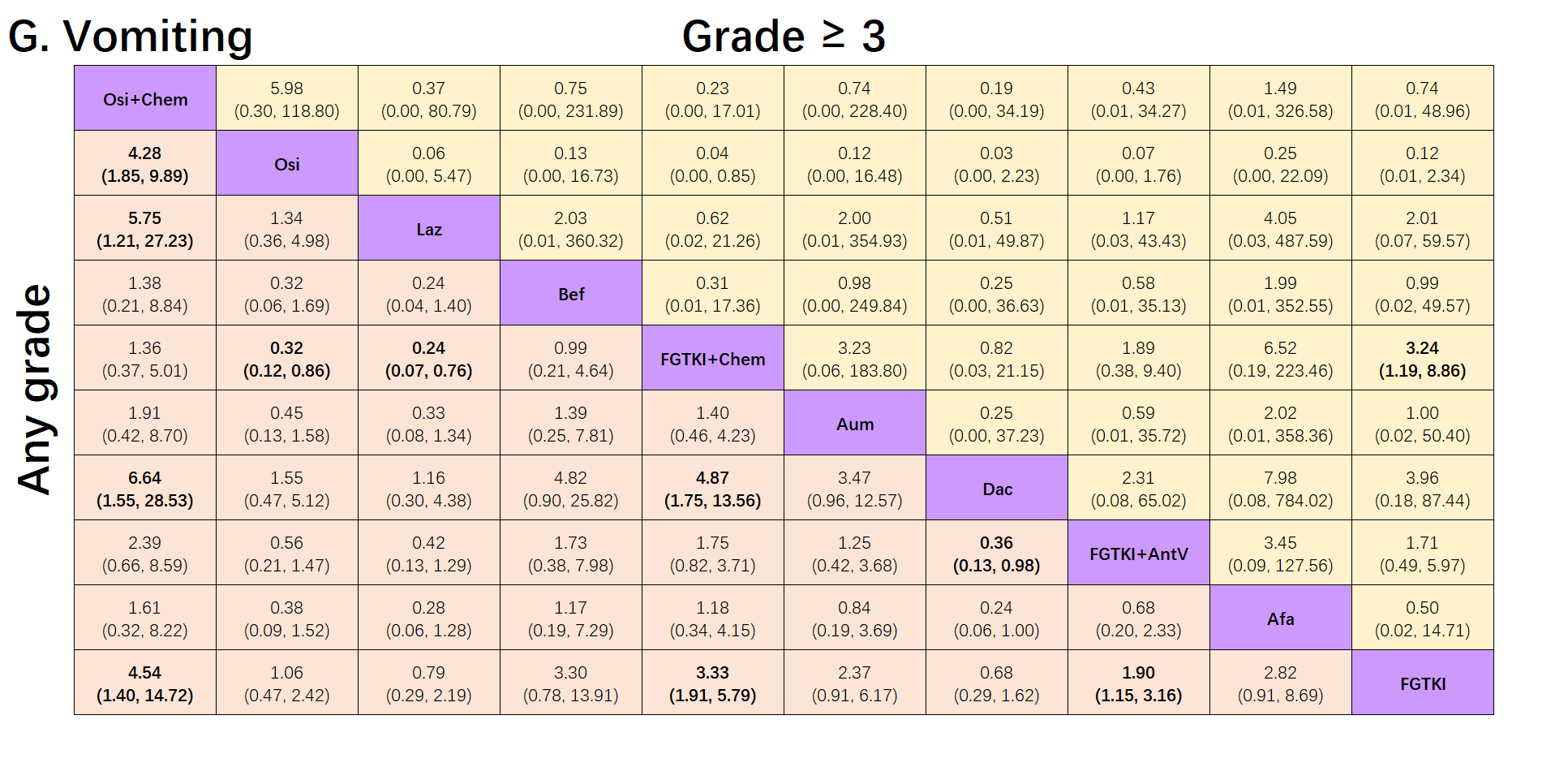
**

**Legend:** The lower triangle represents risk ratios for any grade of vomiting, while the upper triangle represents risk ratios for grade ≥ 3 vomiting. The data in each cell represent risk ratios (95% confidence intervals) comparing the treatment defined in the column with the treatment defined in the row. Significant results are indicated in bold. FGTKI = first-generation EGFR-TKIs; Afa = afatinib; Dac = dacomitinib; Osi = osimertinib; Bef = befotertinib; Laz = lazertinib; Aum = aumolertinib; Cet = cetuximab; Chem = chemotherapy.

**
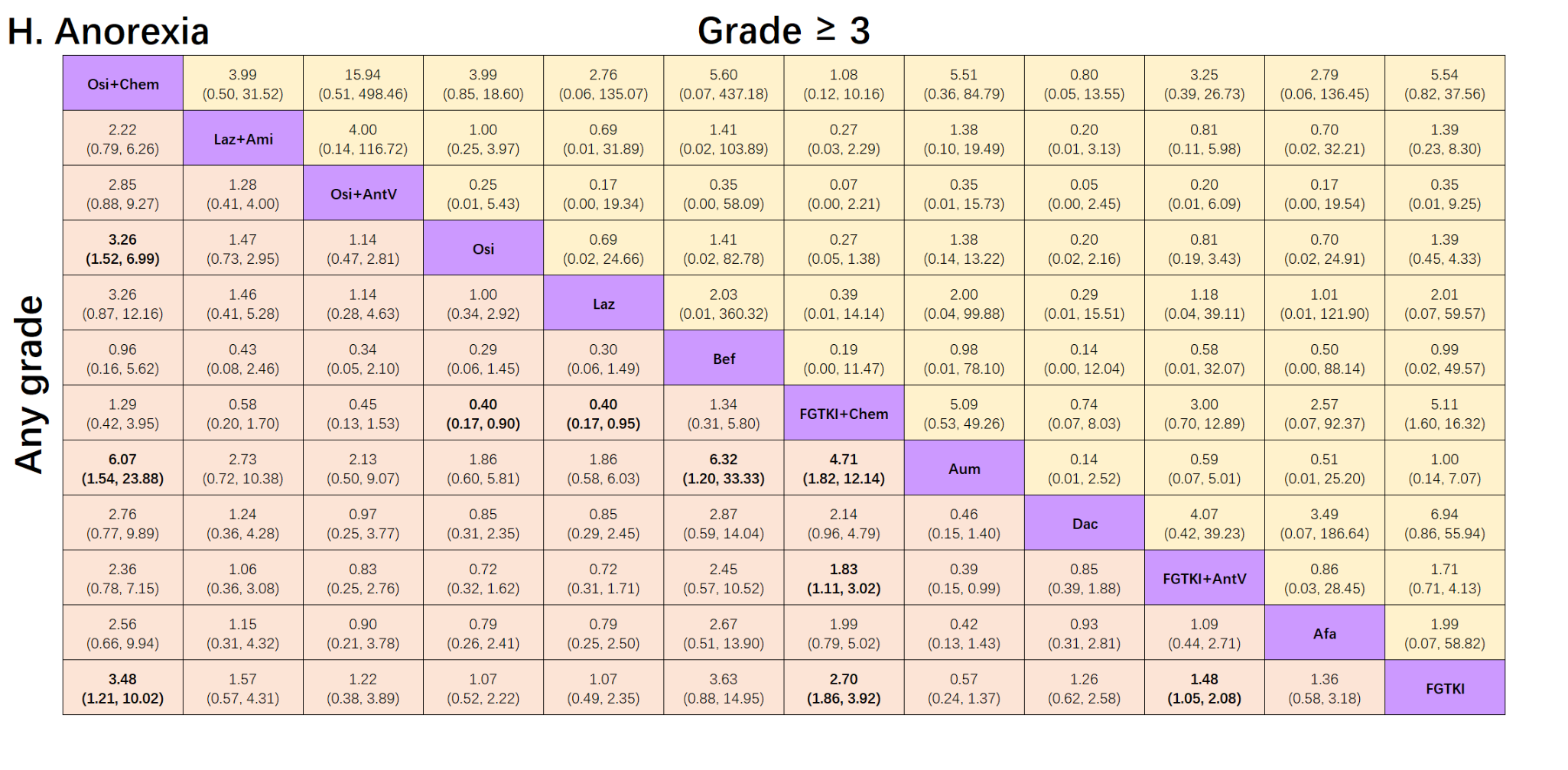
**

**Legend:** The lower triangle represents risk ratios for any grade of anorexia, while the upper triangle represents risk ratios for grade ≥ 3 anorexia.The data in each cell represent risk ratios (95% confidence intervals) comparing the treatment defined in the column with the treatment defined in the row. Significant results are indicated in bold. FGTKI = first-generation EGFR-TKIs; Afa = afatinib; Dac = dacomitinib; Osi = osimertinib; Bef = befotertinib; Laz = lazertinib; Aum = aumolertinib; Ami= amivantamab; Cet = cetuximab; Chem = chemotherapy; AntV = antiangiogenic agents.

**
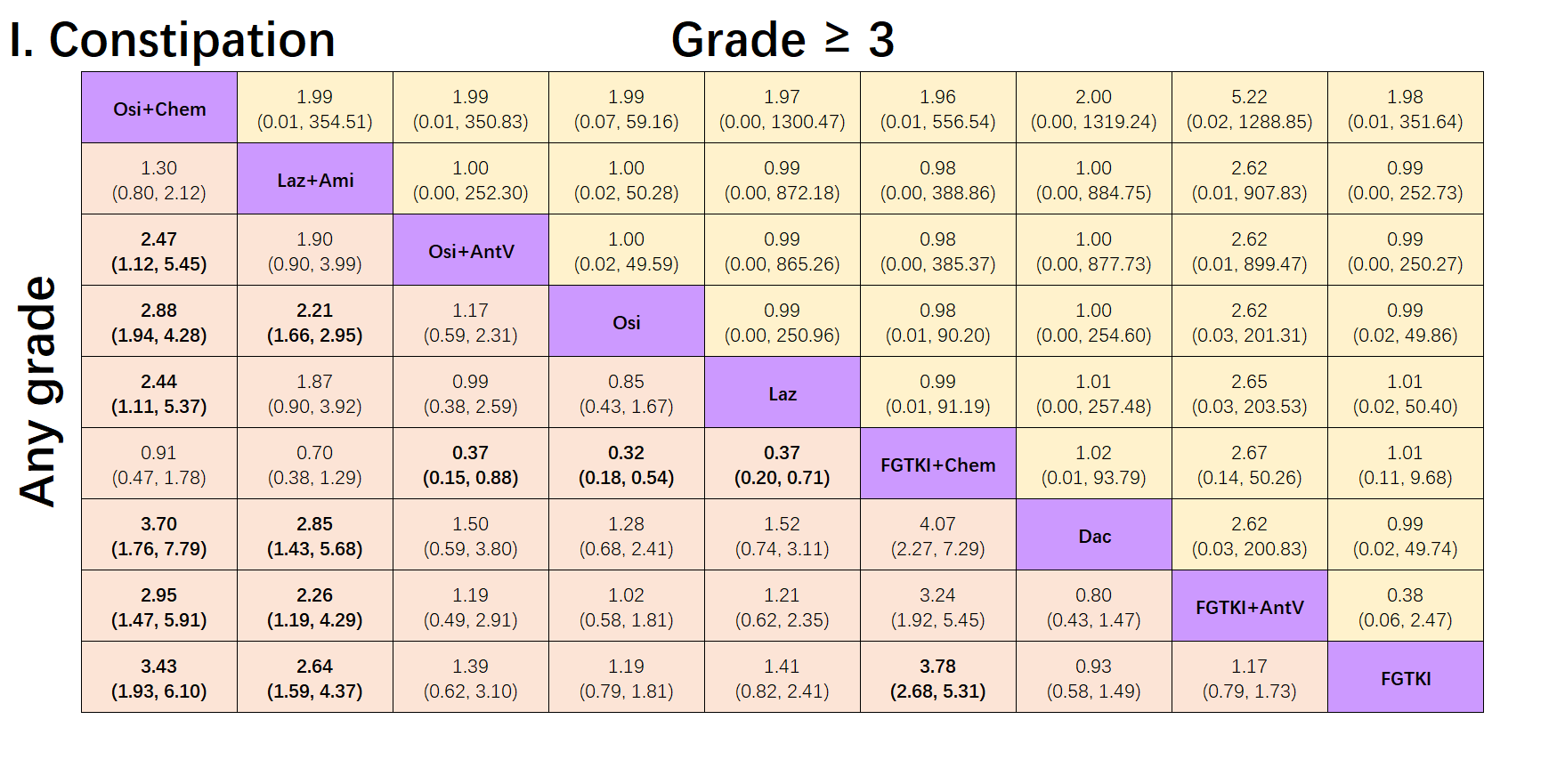
**

**Legend:** The lower triangle represents risk ratios for any grade of constipation, while the upper triangle represents risk ratios for grade ≥ 3 constipation. The data in each cell represent risk ratios (95% confidence intervals) comparing the treatment defined in the column with the treatment defined in the row. Significant results are indicated in bold. FGTKI = first-generation EGFR-TKIs; Dac = dacomitinib; Osi = osimertinib; Laz = lazertinib; Aum = aumolertinib; Ami= amivantamab; Chem = chemotherapy; AntV = antiangiogenic agents.

**
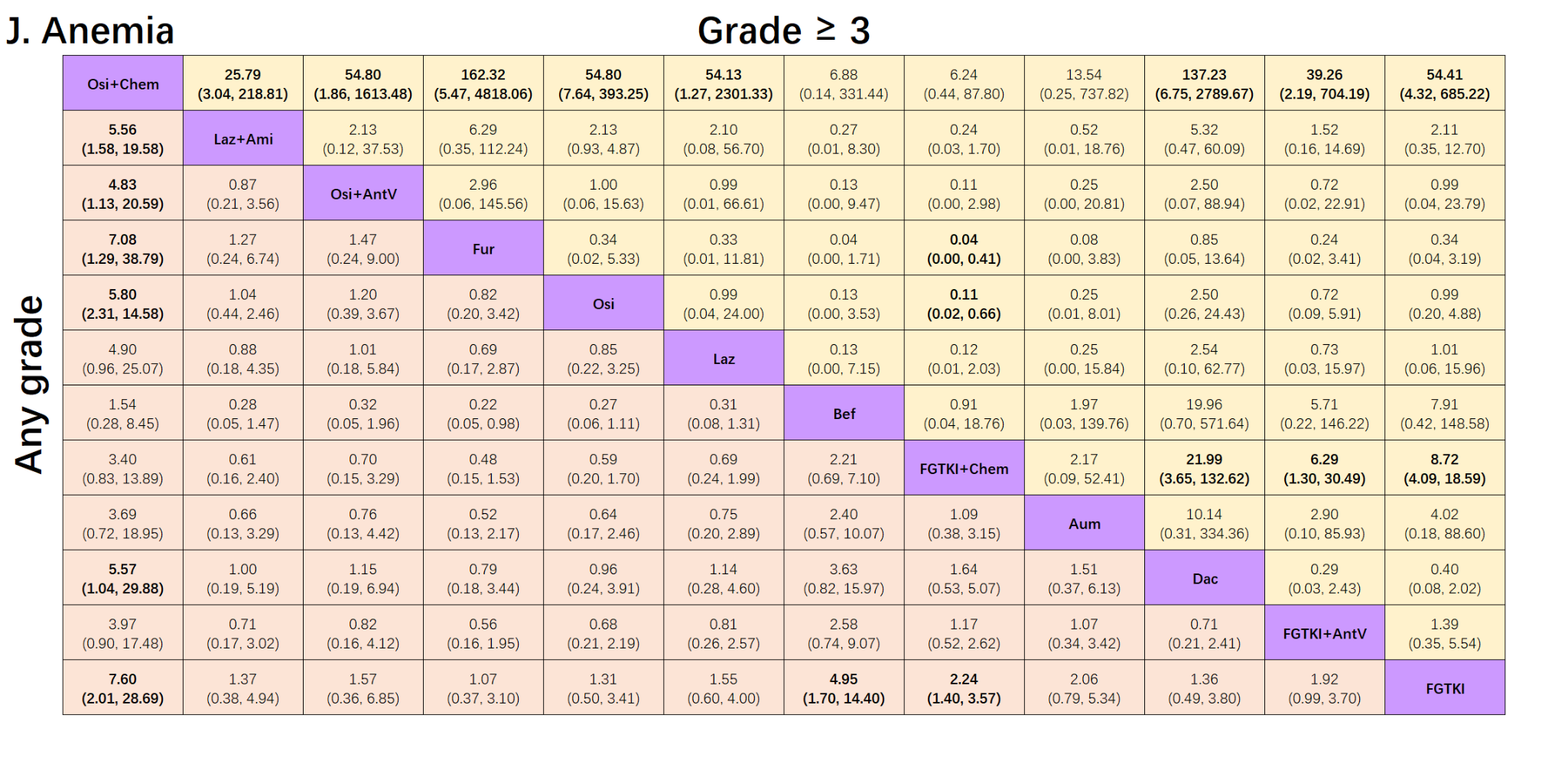
**

**Legend:** The lower triangle represents risk ratios for any grade of anemia, while the upper triangle represents risk ratios for grade ≥ 3 anemia. The data in each cell represent risk ratios (95% confidence intervals) comparing the treatment defined in the column with the treatment defined in the row. Significant results are indicated in bold. FGTKI = first-generation EGFR-TKIs; Afa = afatinib; Dac = dacomitinib; Osi = osimertinib; Bef = befotertinib; Fur = furmonertinib; Laz = lazertinib; Aum = aumolertinib; Ami= amivantamab; Cet = cetuximab; Chem = chemotherapy; AntV = antiangiogenic agents.

**
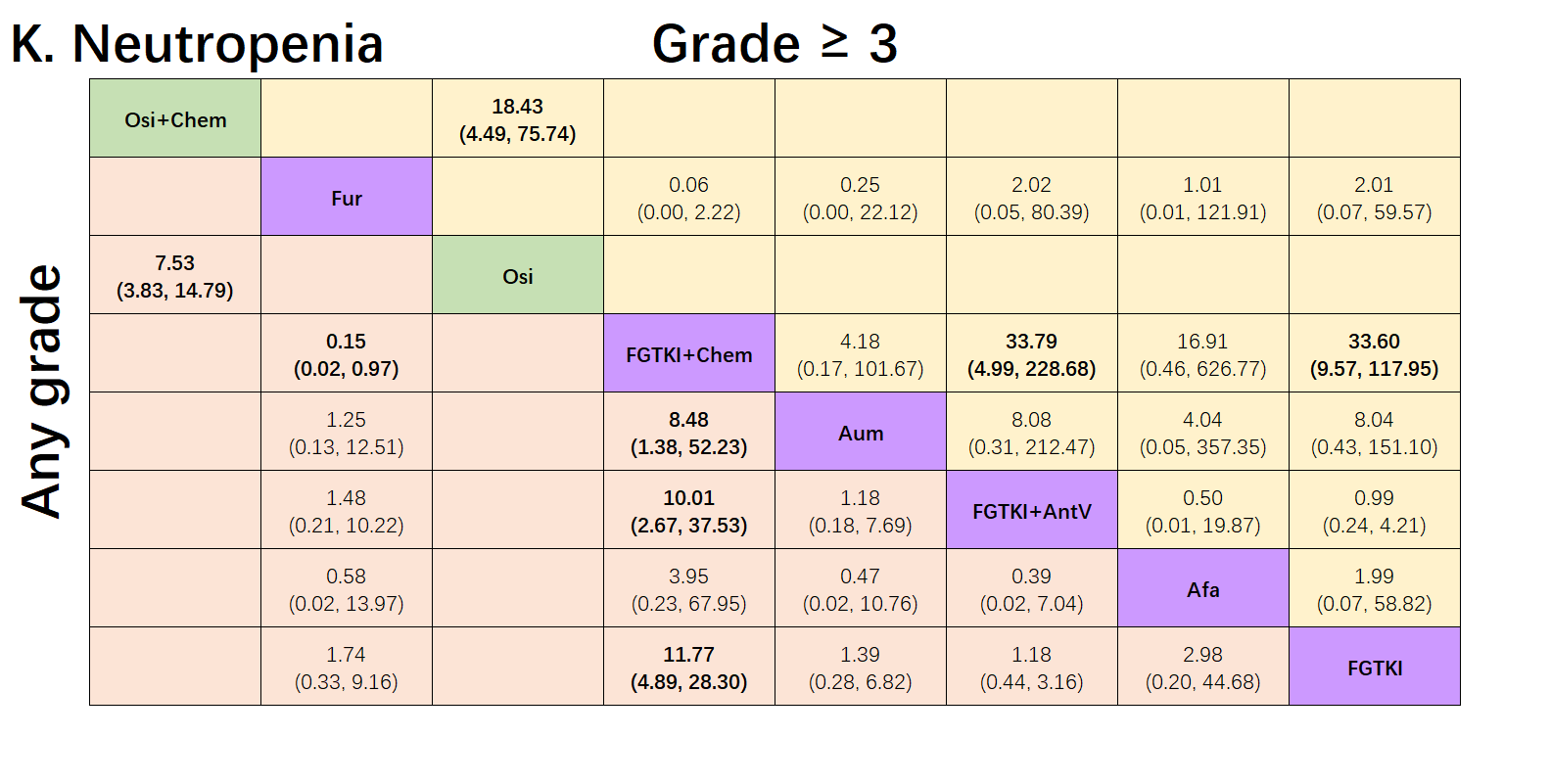
**

**Legend:** The lower triangle represents risk ratios for any grade of neutropenia, while the upper triangle represents risk ratios for grade ≥ 3 neutropenia. The data in each cell represent risk ratios (95% confidence intervals) comparing the treatment defined in the column with the treatment defined in the row. Significant results are indicated in bold. The cell of the treatment group with different background colors means a different loop. FGTKI = first-generation EGFR-TKIs; Afa = afatinib; Osi = osimertinib; Fur = furmonertinib; Aum = aumolertinib; Chem = chemotherapy; AntV = antiangiogenic agents.

**
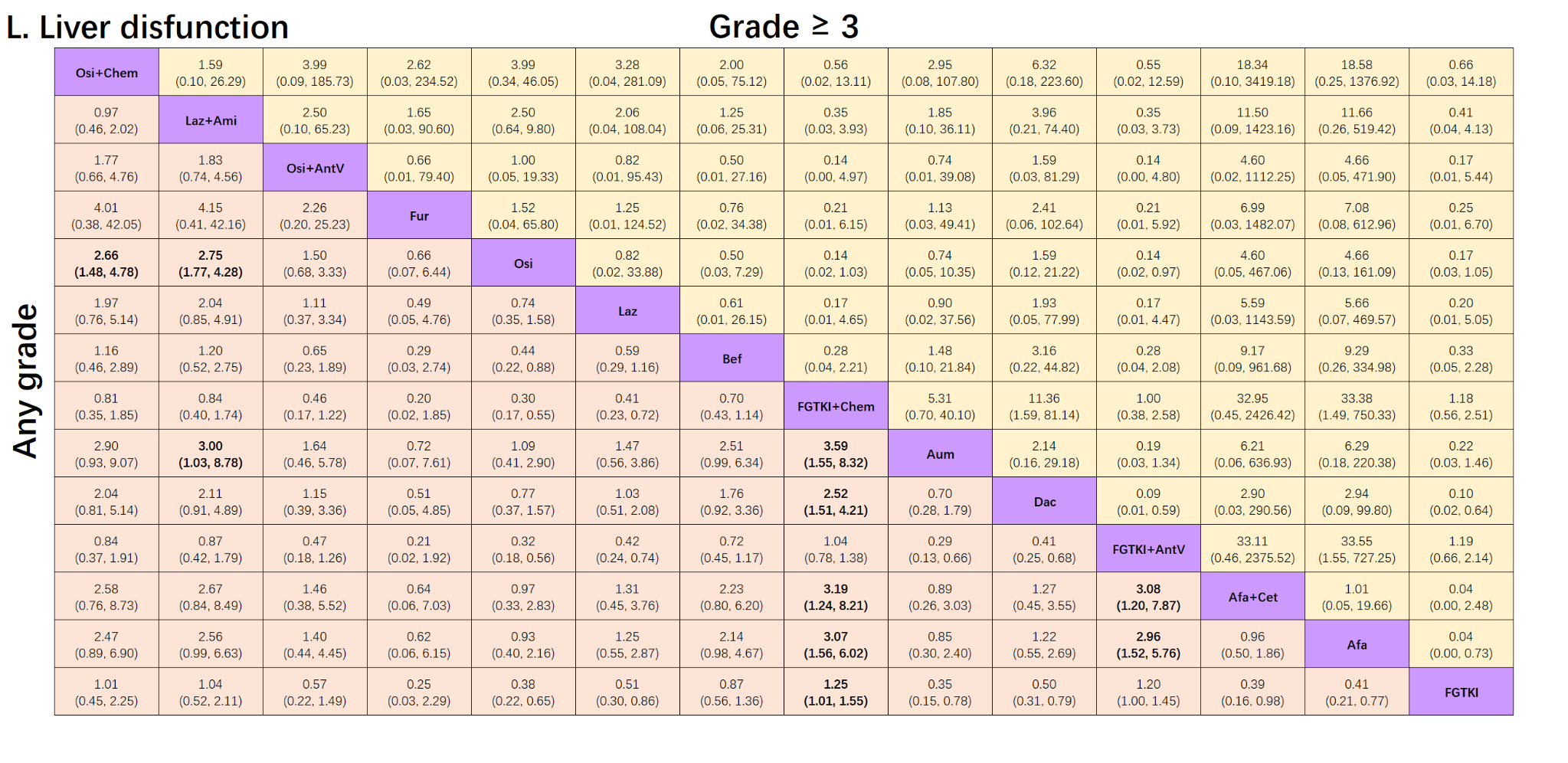
**

**Legend:** The lower triangle represents risk ratios for any grade of liver disfunction, while the upper triangle represents risk ratios for grade ≥ 3 liver disfunction. The data in each cell represent risk ratios (95% confidence intervals) comparing the treatment defined in the column with the treatment defined in the row. Significant results are indicated in bold. FGTKI = first-generation EGFR-TKIs; Afa = afatinib; Dac = dacomitinib; Osi = osimertinib; Bef = befotertinib; Fur = furmonertinib; Laz = lazertinib; Aum = aumolertinib; Ami= amivantamab; Cet = cetuximab; Chem = chemotherapy; AntV = antiangiogenic agents.

**
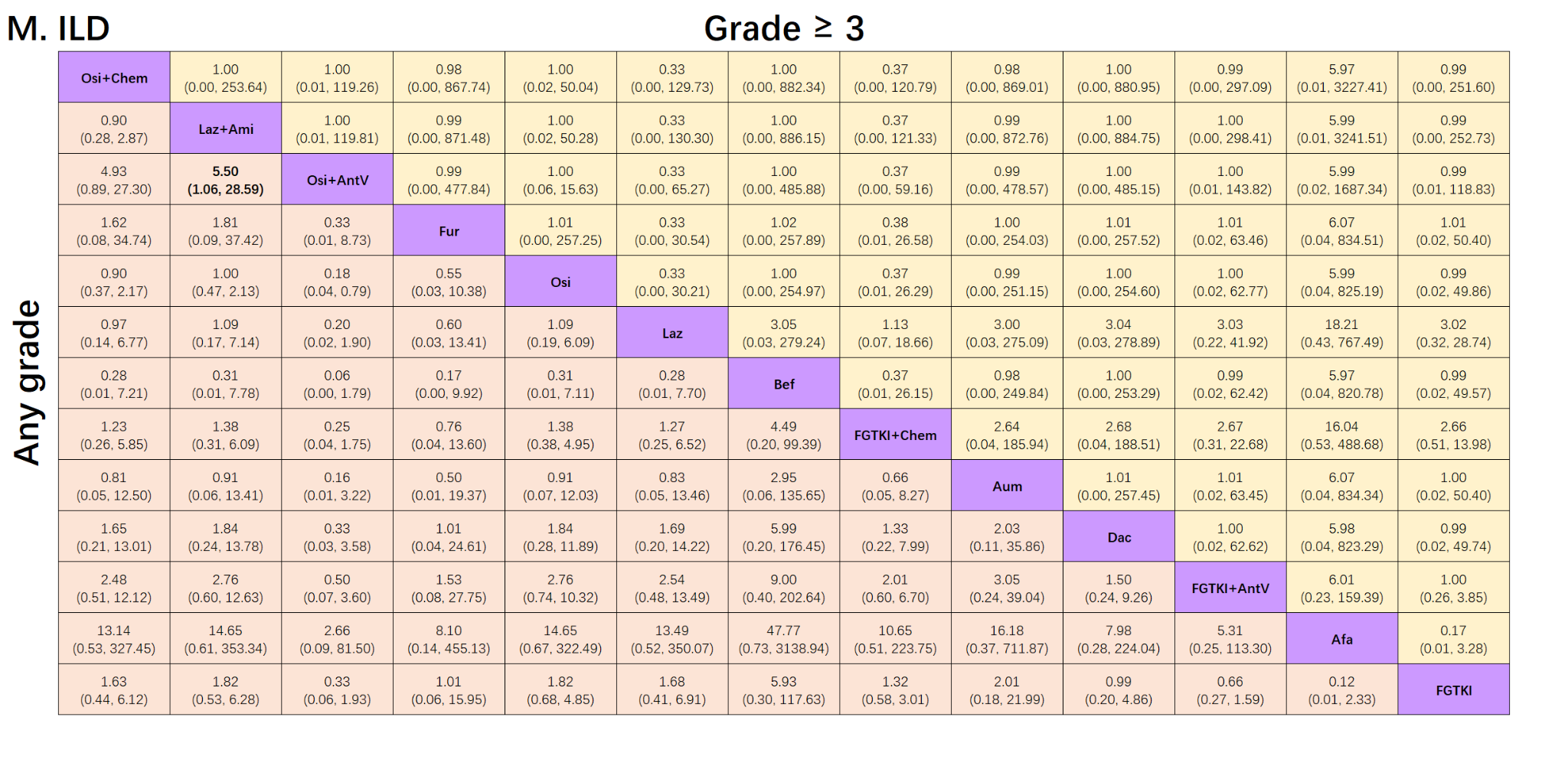
**

**Legend:** The lower triangle represents risk ratios for any grade of proteinuria, while the upper triangle represents risk ratios for grade ≥ 3 proteinuria. The data in each cell represent risk ratios (95% confidence intervals) comparing the treatment defined in the column with the treatment defined in the row. Significant results are indicated in bold. The cell of the treatment group with different background colours means a different loop. FGTKI = first-generation EGFR-TKIs; Osi = osimertinib; Bef = befotertinib; Chem = chemotherapy; AntV = antiangiogenic agents.

**
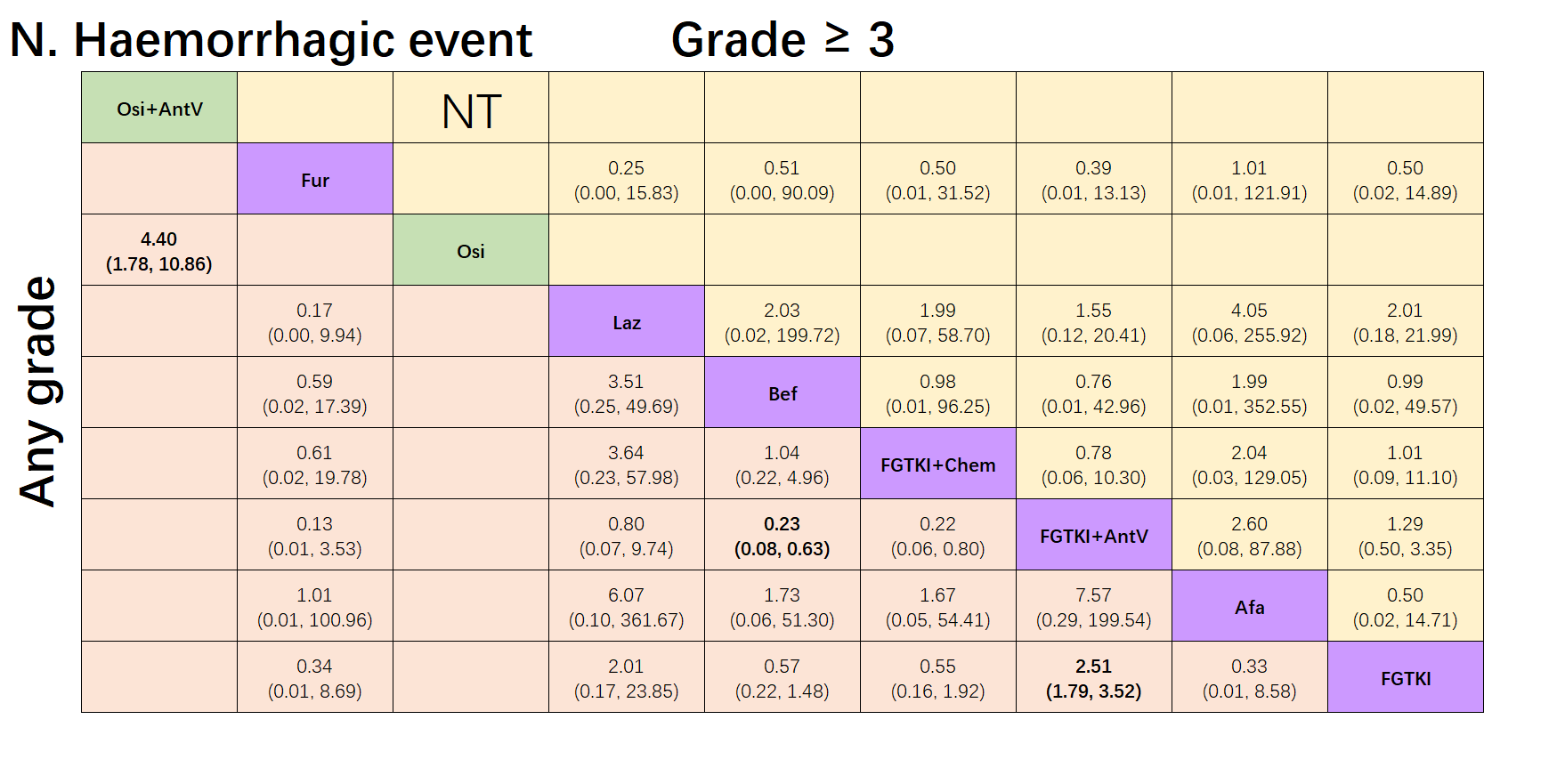
**

**Legend:** The lower triangle represents risk ratios for any grade of haemorrhagic event, while the upper triangle represents risk ratios for grade ≥ 3 haemorrhagic event.The data in each cell represent risk ratios (95% confidence intervals) comparing the treatment defined in the column with the treatment defined in the row. Significant results are indicated in bold. The cell of the treatment group with different background colours means a different loop. FGTKI = first-generation EGFR-TKIs; Afa = afatinib; Osi = osimertinib; Bef = befotertinib; Laz = lazertinib; Chem = chemotherapy; AntV = antiangiogenic agents.

**
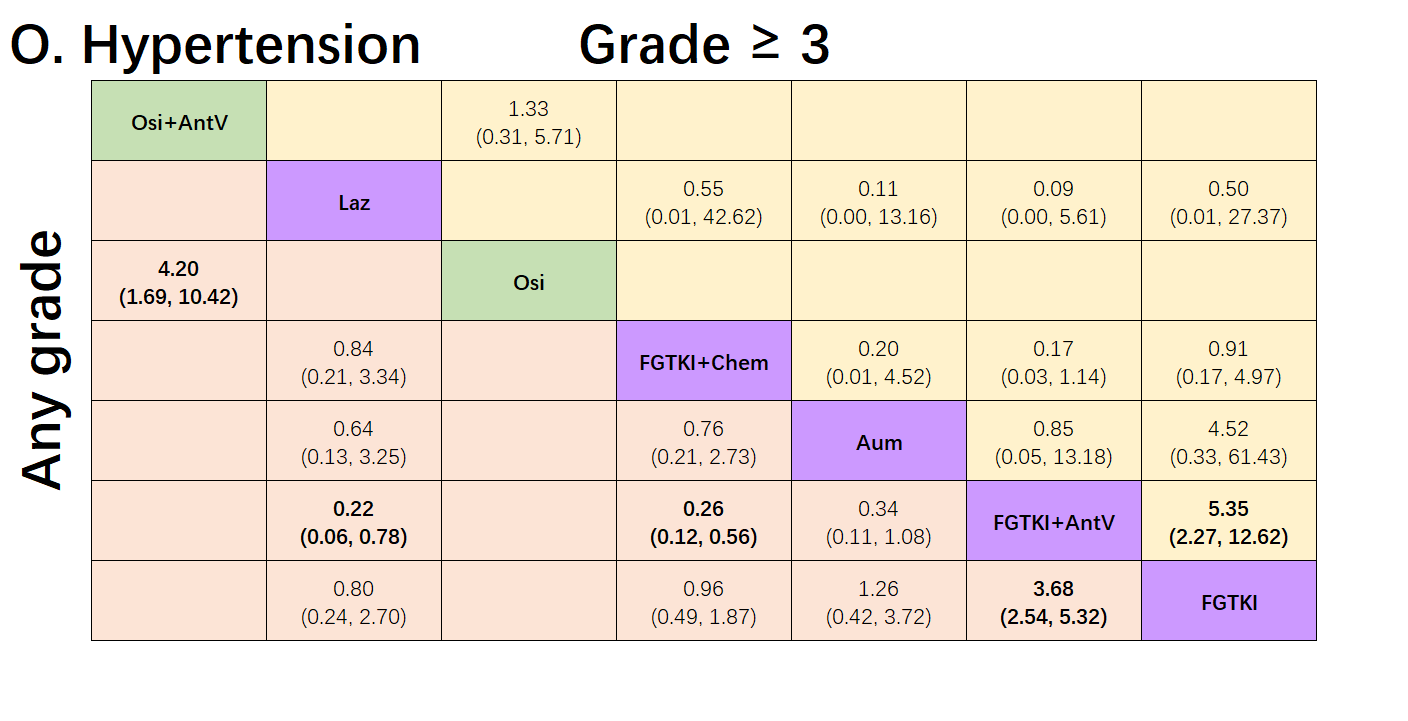
**

**Legend:** The lower triangle represents risk ratios for any grade of hypertension, while the upper triangle represents risk ratios for grade ≥ 3 hypertension. The data in each cell represent risk ratios (95% confidence intervals) comparing the treatment defined in the column with the treatment defined in the row. Significant results are indicated in bold. The cell of the treatment group with different background colours means a different loop. FGTKI = first-generation EGFR-TKIs; Osi = osimertinib; Aum = aumolertinib; Laz = lazertinib; Chem = chemotherapy; AntV = antiangiogenic agents.

**
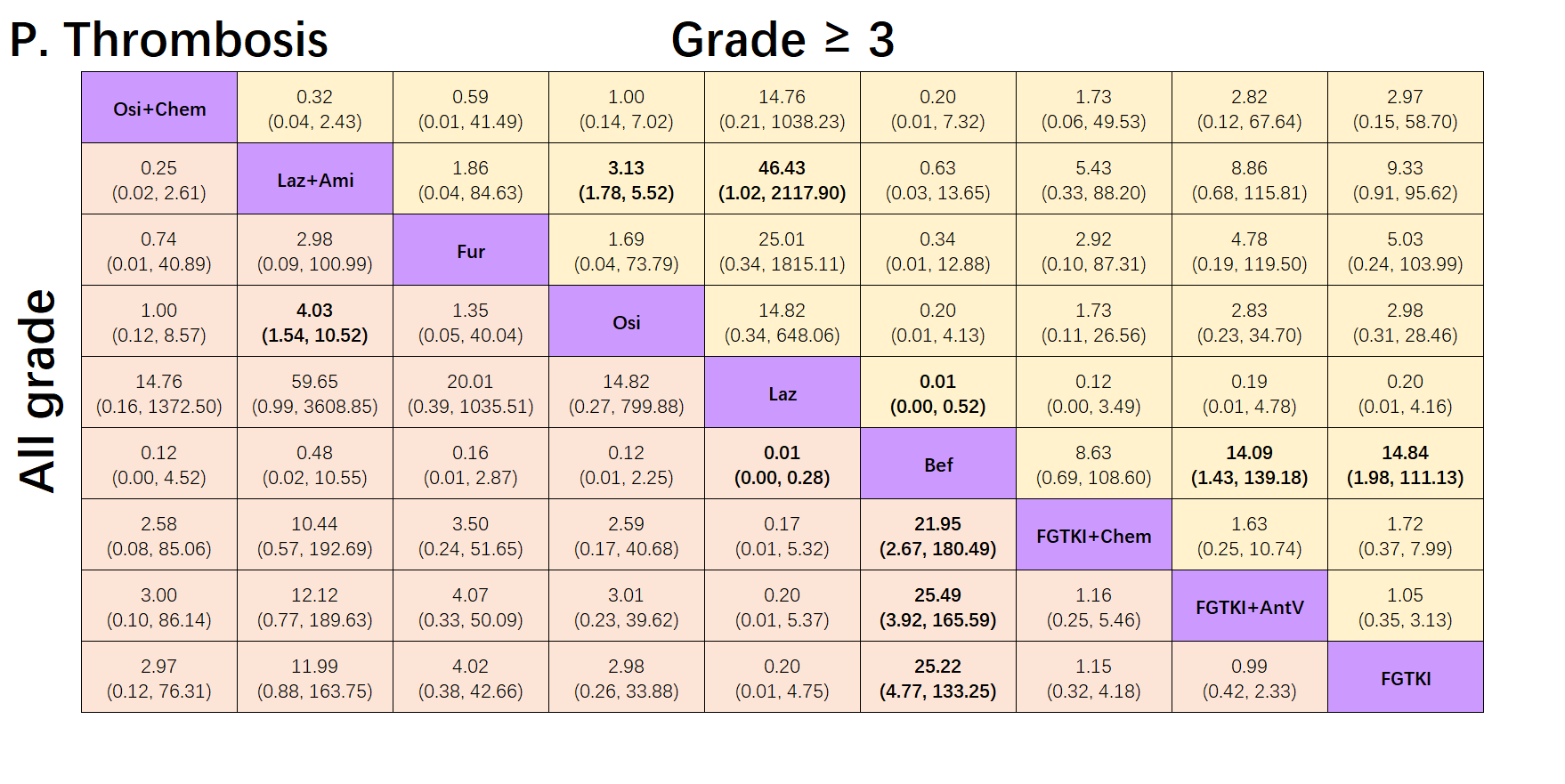
**

**Legend:** The lower triangle represents risk ratios for any grade of thrombosis, while the upper triangle represents risk ratios for grade ≥ 3 thrombosis.The data in each cell represent risk ratios (95% confidence intervals) comparing the treatment defined in the column with the treatment defined in the row. Significant results are indicated in bold. FGTKI = first-generation EGFR-TKIs; Osi = osimertinib; Bef = befotertinib; Fur = furmonertinib; Laz = lazertinib; Aum = aumolertinib; Ami= amivantamab; Chem = chemotherapy; AntV = antiangiogenic agents.

**
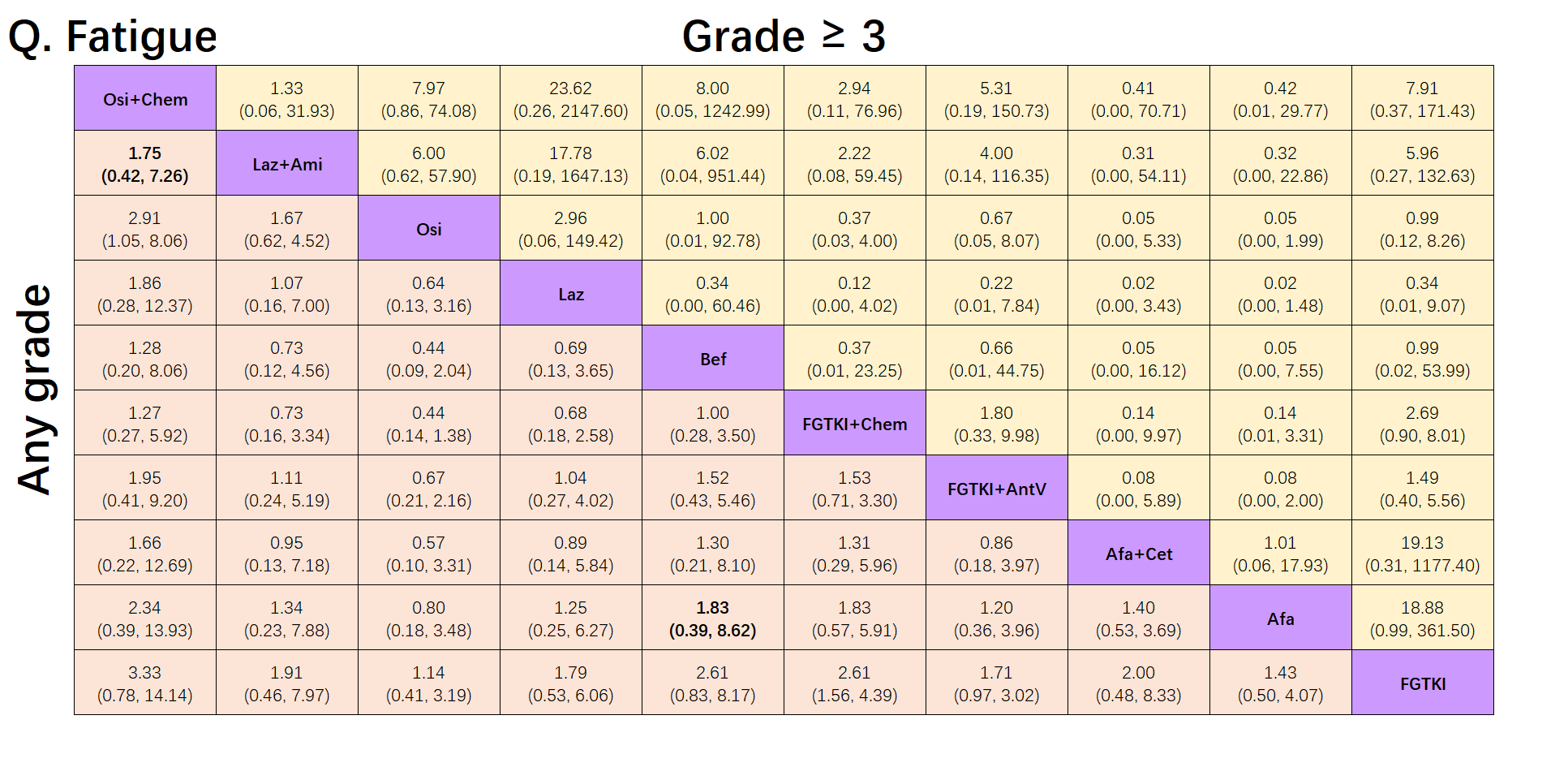
**

**Legend:** The lower triangle represents risk ratios for any grade of fatigue, while the upper triangle represents risk ratios for grade ≥ 3 fatigue.The data in each cell represent risk ratios (95% confidence intervals) comparing the treatment defined in the column with the treatment defined in the row. Significant results are indicated in bold. FGTKI = first-generation EGFR-TKIs; Afa = afatinib; Osi = osimertinib; Bef = befotertinib; Laz = lazertinib; Ami= amivantamab; Cet = cetuximab; Chem = chemotherapy; AntV = antiangiogenic agents.

**
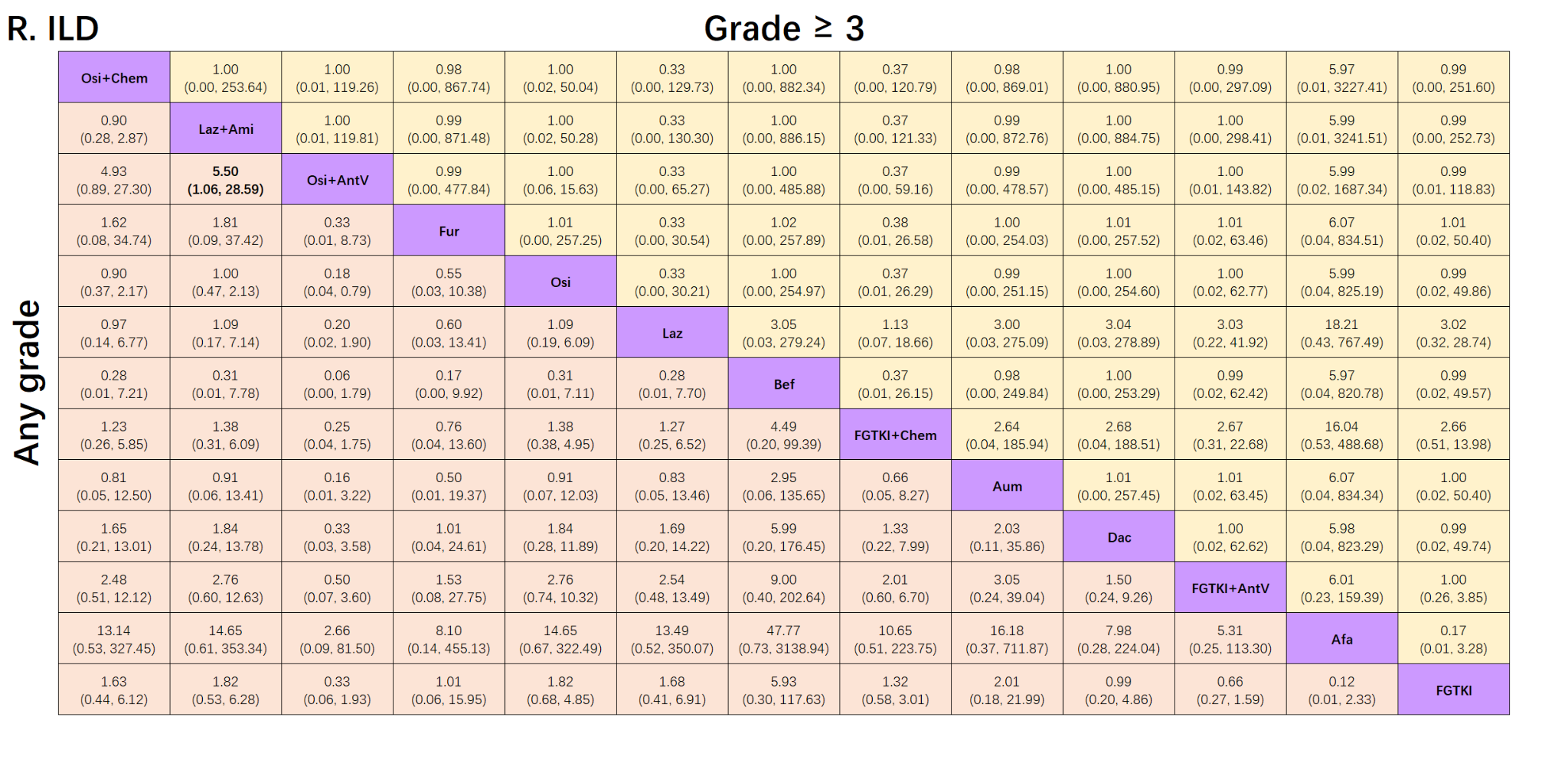
**

**Legend:** The lower triangle represents risk ratios for any grade of ILD, while the upper triangle represents risk ratios for grade ≥ 3 ILD.The data in each cell represent risk ratios (95% confidence intervals) comparing the treatment defined in the column with the treatment defined in the row. Significant results are indicated in bold. ILD= interstitial lung disease; FGTKI = first-generation EGFR-TKIs; Afa = afatinib; Dac = dacomitinib; Osi = osimertinib; Bef = befotertinib; Fur = furmonertinib; Laz = lazertinib; Aum = aumolertinib; Ami= amivantamab; Cet = cetuximab; Chem = chemotherapy; AntV = antiangiogenic agents.

**eFigure 4.** Sensitivity analysis including only phase III trials.

**
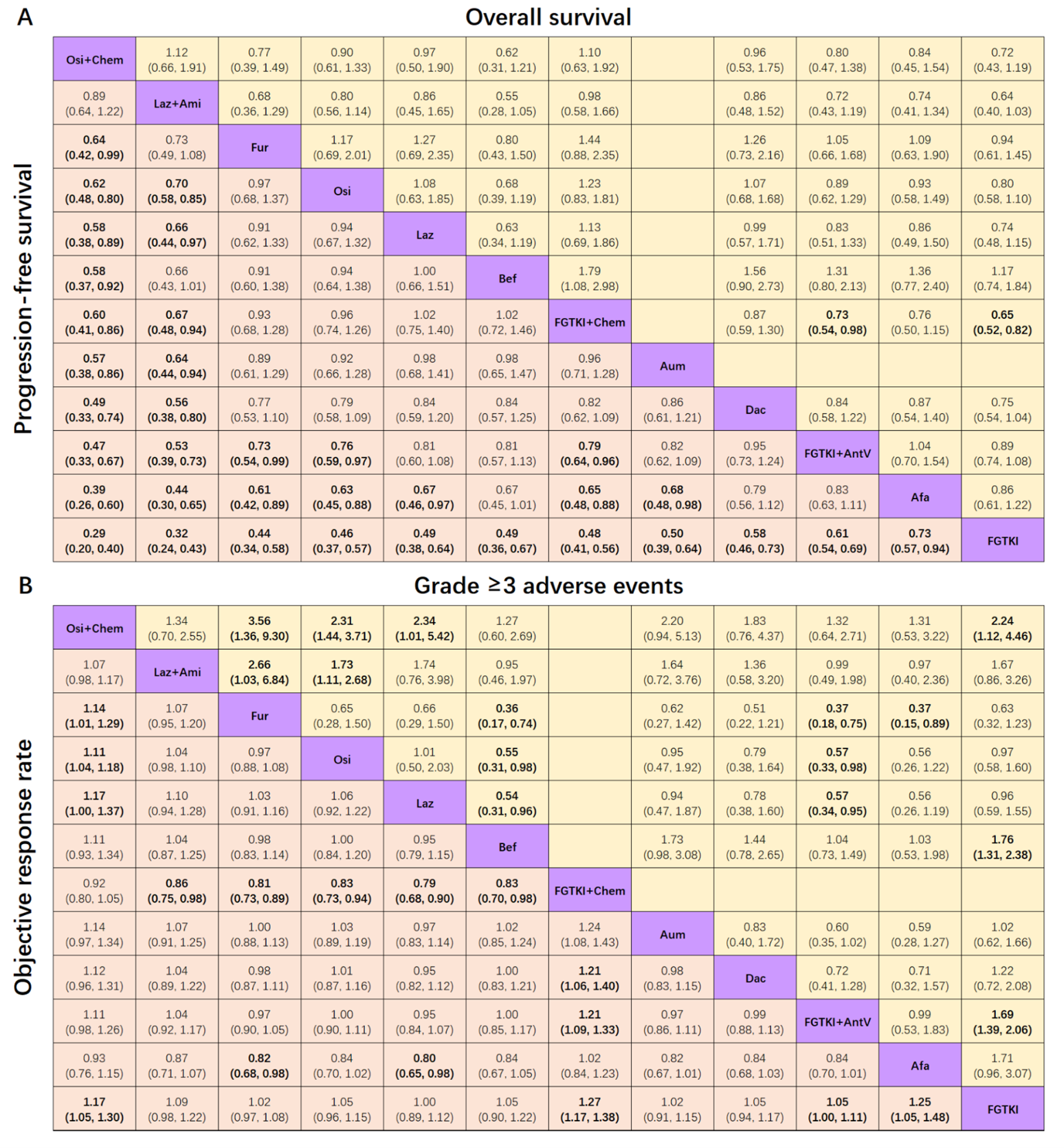
**

**Legend:** A: Pooled hazard ratios (95% confidence intervals) for progression-free survival (lower triangle) and overall survival (upper triangle) in patients with advanced EGFR-mutated NSCLC. B: Pooled risk ratios (95% confidence intervals) for objective response rate (lower triangle) and grade ≥3 adverse events (upper triangle) in patients with advanced EGFR-mutated NSCLC. The data in each cell represent hazard or risk ratios (95% confidence intervals) comparing the treatment defined in the column with the treatment defined in the row. Significant results are indicated in bold.

**eFigure5.** Funnel plots of the network meta-analysis.

**
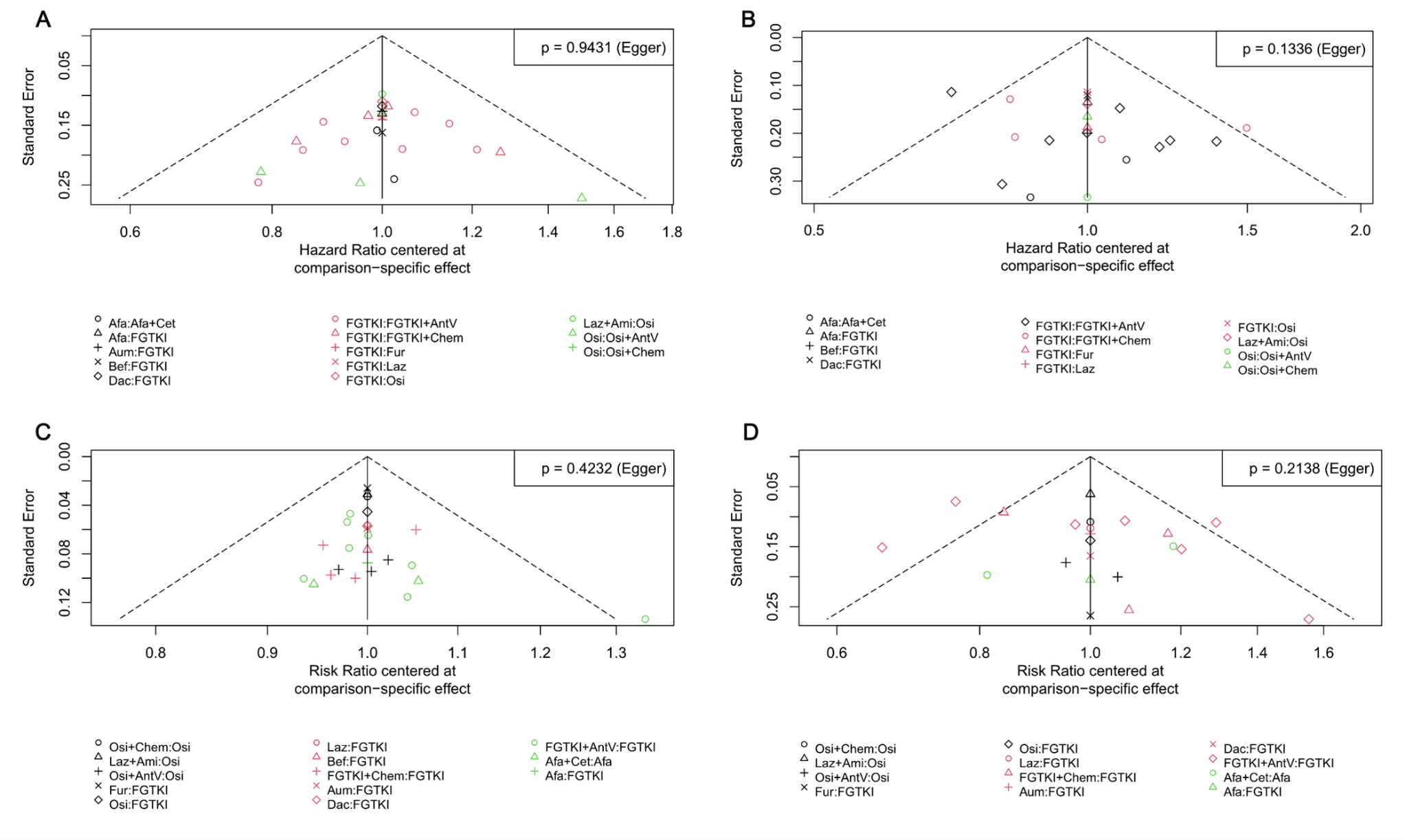
**

**Legend:** A-D: progression-free survival, overall survival, objective response rate and grade ≥ 3 adverse events. FGTKI = first-generation EGFR-TKIs; Afa = afatinib; Dac = dacomitinib; Osi = osimertinib; Bef = befotertinib; Fur = furmonertinib; Laz = lazertinib; Aum = aumolertinib; Ami= amivantamab; Cet = cetuximab; Chem = chemotherapy; AntV = antiangiogenic agents.
